# Supplementary material for: Assessing connectivity despite high diversity in island populations of a malaria mosquito
Source: Evol Appl. 2019 Oct 28;13(2):417–31. doi: 10.1111/eva.12878 (PMC6976967; doi:10.1111/eva.12878)
Supplement: Supplementary file 1 [file EVA-13-417-s001.pdf]

Supplemental Material for: Assessing connectivity despite high diversity in island populations of a malaria mosquito

Christina M. Bergey, Martin Lukindu, Rachel M. Wiltshire, Michael C. Fontaine, Jonathan K. Kayondo, and Nora J. Besansky

Tables

Table S1: Sampling sites and coordinates. Values in parentheses indicate counts per subsample for Bugala sampling sites and the mainland- and island-like subgroups used in analyses.

Location	Latitude	Longitude	Sample Count
Banda	-0.25893	32.39594	11
Bugala - All sites	-	-	23
Bugala - Bugoma	-0.26697	32.07936	(11)
Bugala - Lutoboka	-0.31624	32.29246	(7)
Bugala - Mweena	-0.32806	32.31113	(5)
Bugala - Mainland-like portion	-	-	(12)
Bugala - Island-like portion	-	-	(11)
Bukasa	-0.48609	32.45091	11
Buwama	0.02077	32.10574	11
Kaazi	-0.31831	31.88183	11
Kiyindi	0.27558	33.14699	10
Nsadzi	-0.08632	32.58895	11
Sserinya	-0.26476	32.37228	16
Wamala	0.40811	31.99609	11

Table S2: List of individuals included in study with mean depth of sequencing coverage.

ID	Field ID	Locality	Specific Sampling Site	Mean depth
LVB2015-1	CM-KSB-J5	Nsadzi	Kansambwe	20.40
LVB2015-2	K-KSB-E1	Nsadzi	Kansambwe	24.50
LVB2015-3	RM-KSB-G1	Nsadzi	Kansambwe	17.90
LVB2015-4	NKG-F-G3	Bukasa	Nakibanga	4.90
LVB2015-6	NKG-F-H1	Bukasa	Nakibanga	15.80
LVB2015-7	NKG-K-I1	Bukasa	Nakibanga	15.80
LVB2015-8	NKG-K-K1	Bukasa	Nakibanga	19.90
LVB2015-9	NKG-M-C1	Bukasa	Nakibanga	22.30
LVB2015-10	NKG-M-D1	Bukasa	Nakibanga	20.30
LVB2015-11	NKG-M-F1	Bukasa	Nakibanga	23.90
LVB2015-14	MWN-K-A1	Bugala	Mweena	21.20
LVB2015-15	MWN-K-C2	Bugala	Mweena	18.30
LVB2015-16	MWN-P-D1	Bugala	Mweena	5.34

LVB2015-17	MWN-R-E1	Bugala	Mweena	22.60
LVB2015-18	MWN-R-F1	Bugala	Mweena	17.10
LVB2015-19	BDA-K-B1	Banda	Banda	14.40
LVB2015-20	BDA-K-B2	Banda	Banda	18.00
LVB2015-21	BBS-C-M1	Sserinya	Bbosa	19.70
LVB2015-22	BBS-F-F1	Sserinya	Bbosa	20.80
LVB2015-24	BBS-K-J3	Sserinya	Bbosa	22.80
LVB2015-25	BBS-K-J8	Sserinya	Bbosa	17.30
LVB2015-26	BBS-K-K2	Sserinya	Bbosa	18.20
LVB2015-27	BBS-M-L1	Sserinya	Bbosa	22.40
LVB2015-28	BBS-P-I4	Sserinya	Bbosa	23.30
LVB2015-29	BBS-R-A2	Sserinya	Bbosa	19.60
LVB2015-30	BBS-R-C1	Sserinya	Bbosa	16.10
LVB2015-32	KSS-F-E2	Sserinya	Kasisa	21.00
LVB2015-33	LBK-C-F1	Bugala	Lutoboka	21.00
LVB2015-34	LBK-C-F6	Bugala	Lutoboka	18.60
LVB2015-35	LBK-C-G6	Bugala	Lutoboka	20.90
LVB2015-36	LBK-K-E2	Bugala	Lutoboka	22.00
LVB2015-37	LBK-M-A1	Bugala	Lutoboka	18.50
LVB2015-39	LBK-R-O1	Bugala	Lutoboka	20.20
LVB2015-42	BGM-F-D1	Bugala	Bugoma	16.70
LVB2015-43	BGM-F-E2	Bugala	Bugoma	24.50
LVB2015-45	BGM-K-M2	Bugala	Bugoma	23.80
LVB2015-46	BGM-M-G1	Bugala	Bugoma	18.40
LVB2015-47	BGM-M-H2	Bugala	Bugoma	14.10
LVB2015-48	BGM-M-J1	Bugala	Bugoma	20.90
LVB2015-50	BGM-P-F9	Bugala	Bugoma	18.90
LVB2015-51	BGM-R-O2	Bugala	Bugoma	16.80
LVB2015-52	KZI-F-F001	Kaazi	Nabugabo	19.10
LVB2015-53	KZI-F-G001	Kaazi	Nabugabo	19.50
LVB2015-54	KZI-F-H001	Kaazi	Nabugabo	19.40
LVB2015-55	KZI-P-A001	Kaazi	Nabugabo	10.30
LVB2015-56	KZI-P-B005	Kaazi	Nabugabo	16.50
LVB2015-59	KZI-R-C003	Kaazi	Nabugabo	18.10
LVB2015-60	KZI-R-D007	Kaazi	Nabugabo	15.90
LVB2015-61	BWM-C-G001	Buwama	Buwama	16.10
LVB2015-62	BWM-C-H001	Buwama	Buwama	11.10

LVB2015-63	BWM-F-A001	Buwama	Buwama	20.40
LVB2015-64	BWM-F-B001	Buwama	Buwama	21.50
LVB2015-65	BWM-P-J001	Buwama	Buwama	14.80
LVB2015-66	BWM-R-C002	Buwama	Buwama	19.10
LVB2015-67	BWM-R-F005	Buwama	Buwama	22.30
LVB2015-68	NMA-C-E003	Wamala	Naama	20.30
LVB2015-69	NMA-C-F002	Wamala	Naama	17.80
LVB2015-70	NMA-F-A001	Wamala	Naama	13.10
LVB2015-71	NMA-K-B001	Wamala	Naama	22.10
LVB2015-72	NMA-K-C002	Wamala	Naama	18.00
LVB2015-73	NMA-P-G001	Wamala	Naama	18.20
LVB2015-74	NMA-P-H003	Wamala	Naama	16.60
LVB2015-76	KYD-C-G001	Kiyindi	Kiyindi	16.10
LVB2015-77	KYD-C-H001	Kiyindi	Kiyindi	11.80
LVB2015-78	KYD-C-I001	Kiyindi	Kiyindi	16.40
LVB2015-79	KYD-C-J002	Kiyindi	Kiyindi	11.50
LVB2015-80	KYD-F-A003	Kiyindi	Kiyindi	10.30
LVB2015-81	KYD-F-B004	Kiyindi	Kiyindi	21.50
LVB2015-82	KYD-K-D002	Kiyindi	Kiyindi	18.40
LVB2015-84	KYD-R-K001	Kiyindi	Kiyindi	16.80
LVB2015-89	BDA-K-E2	Banda	Banda	15.10
LVB2015-90	BDA-K-F1	Banda	Banda	25.10
LVB2015-91	BDA-M-N1	Banda	Banda	25.60
LVB2015-92	BDA-M-O4	Banda	Banda	17.60
LVB2015-93	BDA-M-Q1	Banda	Banda	39.20
LVB2015-96	CM-KSB-J2	Nsadzi	Kansambwe	9.22
LVB2015-97	CM-KSB-J3	Nsadzi	Kansambwe	10.10
LVB2015-98	CM-KSB-J6	Nsadzi	Kansambwe	16.90
LVB2015-100	K-KSB-D1	Nsadzi	Kansambwe	6.05
LVB2015-101	ML-KSB-M1	Nsadzi	Kansambwe	4.27
LVB2015-102	ML-KSB-M2	Nsadzi	Kansambwe	19.90
LVB2015-103	RM-KSB-G2	Nsadzi	Kansambwe	14.20
LVB2015-104	RM-KSB-G3	Nsadzi	Kansambwe	17.50
LVB2015-105	NKG-R-A12	Bukasa	Nakibanga	15.30
LVB2015-106	NKG-C-E1	Bukasa	Nakibanga	16.20
LVB2015-108	NKG-K-C5	Bukasa	Nakibanga	18.50
LVB2015-109	NKG-M-A1	Bukasa	Nakibanga	12.80

LVB2015-112	BDA-K-D4	Banda	Banda	12.70
LVB2015-113	BDA-K-E3	Banda	Banda	12.20
LVB2015-114	BDA-M-N5	Banda	Banda	15.00
LVB2015-115	BDA-M-P1	Banda	Banda	16.80
LVB2015-116	BBS-C-M3	Sserinya	Bbosa	16.60
LVB2015-117	BBS-K-J1	Sserinya	Bbosa	18.80
LVB2015-118	BBS-K-J11	Sserinya	Bbosa	14.60
LVB2015-120	BBS-K-K6	Sserinya	Bbosa	18.10
LVB2015-121	BBS-P-I8	Sserinya	Bbosa	15.00
LVB2015-122	BBS-R-A19	Sserinya	Bbosa	15.50
LVB2015-125	LBK-R-A5	Bugala	Lutoboka	18.10
LVB2015-126	BGM-K-K1	Bugala	Bugoma	15.20
LVB2015-128	BGM-M-H4	Bugala	Bugoma	20.30
LVB2015-129	BGM-P-F4	Bugala	Bugoma	19.00
LVB2015-130	KZI-F-G005	Kaazi	Nabugabo	18.60
LVB2015-131	KZI-P-A007	Kaazi	Nabugabo	15.80
LVB2015-132	KZI-R-C012	Kaazi	Nabugabo	15.10
LVB2015-133	KZI-R-E011	Kaazi	Nabugabo	16.30
LVB2015-134	BWM-P-I001	Buwama	Buwama	18.20
LVB2015-135	BWM-P-K002	Buwama	Buwama	19.30
LVB2015-136	BWM-R-D001	Buwama	Buwama	14.40
LVB2015-137	BWM-R-F002	Buwama	Buwama	19.90
LVB2015-138	NMA-C-E006	Wamala	Naama	21.90
LVB2015-139	NMA-C-F003	Wamala	Naama	20.40
LVB2015-140	NMA-P-G003	Wamala	Naama	18.90
LVB2015-141	NMA-R-I001	Wamala	Naama	14.10
LVB2015-142	KYD-F-B006	Kiyindi	Kiyindi	18.10
LVB2015-143	KYD-K-E003	Kiyindi	Kiyindi	14.20

Table S3: Results of two population demographic inference with IM model in $\delta a \delta i$ when comparing island to island localities. Numbers in parentheses are bounds of 95% confidence interval computed using Fisher information matrix and 100 bootstrap replicates of 1 Mb from the dataset.

Localities	N_a	% Pop. 1 in Split	Pop. 1 ν_F	Pop. 2 ν_F	Time since split	m_{12}	m_{21}
Banda - Bugala (I)	531,000	0.603	1.94	9,800	3,290	None	None
	(530,000, 532,000)	(0.596, 0.609)	(1.8, 2.09)	(7,060, 12,500)	(3,190, 3,390)		
Banda - Bukasa	526,000	0.568	14.7	9,080	7,580	None	None
	(525,000, 527,000)	(0.556, 0.581)	(13.7, 15.6)	(7,240, 10,900)	(7,470, 7,690)		
Banda - Nsadzi	527,000	0.518	47.1	9,880	9,340	None	None
	(526,000, 528,000)	(0.502, 0.534)	(39.9, 54.4)	(7,310, 12,400)	(9,160, 9,530)		
Banda - Sserinya	531,000	0.489	10.5	9,840	4,430	None	None
	(530,000, 532,000)	(0.464, 0.514)	(8.69, 12.4)	(7,390, 12,300)	(4,250, 4,610)		
Bugala (I) - Bukasa	527,000	0.49	9,840	536	5,290	None	None
	(526,000, 528,000)	(0.471, 0.509)	(7,850, 11,800)	(447, 624)	(5,170, 5,410)		
Bugala (I) - Nsadzi	526,000	0.56	8,980	128	6,680	None	None
	(525,000, 527,000)	(0.536, 0.583)	(6,920, 11,000)	(110, 146)	(6,440, 6,910)		
Bugala (I) - Sserinya	530,000	0.61	5,850	49	2,130	None	None
	(529,000, 531,000)	(0.571, 0.65)	(3,610, 8,090)	(38.3, 59.6)	(1,950, 2,300)		
Bukasa - Nsadzi	527,000	0.499	3,420	335	9,340	None	None
	(526,000, 528,000)	(0.49, 0.507)	(2,860, 3,980)	(293, 377)	(9,190, 9,500)		
Bukasa - Sserinya	525,000	0.503	9,840	6,760	8,930	None	None
	(524,000, 526,000)	(0.495, 0.51)	(7,490, 12,200)	(5,630, 7,880)	(8,780, 9,080)		
Nsadzi - Sserinya	540,000	0.538	893	9,900	9,540	None	None
	(539,000, 541,000)	(0.521, 0.554)	(612, 1,170)	(7,810, 12,000)	(9,280, 9,790)		

Table S4: Results of two population demographic inference with IM model in $\delta a \delta i$ when comparing island to mainland localities. Numbers in parentheses are bounds of 95% confidence interval computed using Fisher information matrix and 100 bootstrap replicates of 1 Mb from the dataset.

Localities	N_a	% Pop. 1 in Split	Pop. 1 ν_F	Pop. 2 ν_F	Time since split	m_{12}	m_{21}
Banda - Bugala (M)	522,000	0.599	3.83	9,900	5,400	None	None
	(521,000, 523,000)	(0.586, 0.612)	(3.59, 4.07)	(8,410, 11,400)	(5,300, 5,500)		
Banda - Buwama	522,000	0.51	1.09	8,520	3,040	None	None
	(521,000, 523,000)	(0.491, 0.529)	(1.01, 1.16)	(6,830, 10,200)	(2,940, 3,150)		
Banda - Kaazi	522,000	0.563	3.99	9,960	5,890	None	None
	(521,000, 523,000)	(0.55, 0.575)	(3.71, 4.28)	(8,370, 11,500)	(5,760, 6,030)		
Banda - Kiyindi	510,000	0.568	1.72	9,910	3,910	None	None
	(509,000, 511,000)	(0.56, 0.577)	(1.69, 1.75)	(8,170, 11,600)	(3,880, 3,950)		
Banda - Wamala	521,000	0.562	3.9	7,970	5,510	None	None
	(520,000, 522,000)	(0.554, 0.57)	(3.66, 4.15)	(6,790, 9,140)	(5,390, 5,620)		
Bugala (I) - Bugala (M)	522,000	0.555	1,440	9,710	4,170	None	None
	(521,000, 523,000)	(0.543, 0.566)	(954, 1,930)	(7,420, 12,000)	(4,040, 4,300)		
Bugala (I) - Buwama	523,000	0.499	0.215	130	190	None	None
	(522,000, 524,000)	(0.496, 0.503)	(0.209, 0.222)	(2.12, 258)	(188, 192)		
Bugala (I) - Kaazi	523,000	0.366	1,420	8,510	5,060	None	None
	(522,000, 524,000)	(0.348, 0.384)	(1,210, 1,620)	(6,610, 10,400)	(4,890, 5,230)		
Bugala (I) - Kiyindi	508,000	0.363	1,350	5,910	3,580	None	None
	(507,000, 509,000)	(0.342, 0.383)	(1,070, 1,620)	(4,470, 7,350)	(3,420, 3,740)		
Bugala (I) - Wamala	520,000	0.486	2,060	9,270	3,700	None	None
	(519,000, 521,000)	(0.462, 0.51)	(1,610, 2,500)	(6,790, 11,800)	(3,570, 3,830)		
Bugala (M) - Bukasa	521,000	0.53	9,000	66.3	4,830	None	None
	(520,000, 522,000)	(0.508, 0.552)	(6,720, 11,300)	(52.2, 80.5)	(4,670, 4,990)		
Bugala (M) - Nsadzi	535,000	0.439	9,220	7.59	4,500	None	None

	(534,000, 536,000)	(0.423, 0.455)	(7,500, 10,900)	(6.91, 8.26)	(4,390, 4,620)		
Bugala (M) - Sserinya	534,000	0.516	9,590	48.8	4,040	None	None
	(533,000, 535,000)	(0.497, 0.535)	(7,640, 11,500)	(42.4, 55.2)	(3,930, 4,160)		
Bukasa - Buwama	522,000	0.501	1.43	9,960	1,690	None	None
	(521,000, 523,000)	(0.496, 0.506)	(1.4, 1.46)	(7,250, 12,700)	(1,670, 1,710)		
Bukasa - Kaazi	522,000	0.501	41.5	9,410	5,490	None	None
	(521,000, 522,000)	(0.491, 0.51)	(33.8, 49.3)	(6,990, 11,800)	(5,320, 5,650)		
Bukasa - Kiyindi	508,000	0.361	18.6	9,220	3,360	None	None
	(507,000, 509,000)	(0.336, 0.386)	(15.8, 21.4)	(6,790, 11,600)	(3,190, 3,540)		
Bukasa - Wamala	520,000	0.39	304	8,060	5,200	None	None
	(519,000, 521,000)	(0.372, 0.409)	(257, 351)	(6,270, 9,850)	(5,050, 5,360)		
Buwama - Nsadzi	524,000	0.54	9,810	1.25	1,930	None	None
	(523,000, 525,000)	(0.502, 0.578)	(6,950, 12,700)	(1.07, 1.44)	(1,800, 2,070)		
Buwama - Sserinya	523,000	0.493	54.2	0.137	187	None	None
	(522,000, 524,000)	(0.488, 0.498)	(24.2, 84.2)	(0.134, 0.14)	(186, 189)		
Kaazi - Nsadzi	524,000	0.489	9,540	19.4	5,730	None	None
	(523,000, 525,000)	(0.47, 0.507)	(7,690, 11,400)	(16, 22.8)	(5,540, 5,910)		
Kaazi - Sserinya	523,000	0.598	6,980	104	4,550	None	None
	(522,000, 524,000)	(0.579, 0.618)	(5,440, 8,510)	(89.7, 119)	(4,410, 4,680)		
Kiyindi - Nsadzi	511,000	0.499	9,940	2.62	2,840	None	None
	(510,000, 512,000)	(0.491, 0.507)	(7,340, 12,500)	(2.26, 2.98)	(2,690, 2,990)		
Kiyindi - Sserinya	509,000	0.5	1,870	0.129	185	None	None
	(508,000, 510,000)	(0.496, 0.503)	(-5,010, 8,750)	(0.126, 0.132)	(183, 187)		
Wamala - Nsadzi	523,000	0.473	8,490	9.72	4,420	None	None
	(522,000, 524,000)	(0.454, 0.492)	(6,720, 10,200)	(8.57, 10.9)	(4,290, 4,560)		
Wamala - Sserinya	521,000	0.649	9,840	39.1	2,790	None	None
	(520,000, 522,000)	(0.643, 0.655)	(7,070, 12,600)	(38.1, 40.1)	(2,760, 2,820)		

Table S5: Results of two population demographic inference with IM model in $\delta a \delta i$ when comparing mainland to mainland localities. Numbers in parentheses are bounds of 95% confidence interval computed using Fisher information matrix and 100 bootstrap replicates of 1 Mb from the dataset.

Localities	N_a	% Pop. 1 in Split	Pop. 1 ν_F	Pop. 2 ν_F	Time since split	m_{12}	m_{21}
Bugala (M) - Buwama	523,000	0.498	9,590	1,090	368	None	None
	(523,000, 524,000)	(0.49, 0.506)	(802, 18,400)	(573, 1,620)	(320, 416)		
Bugala (M) - Kaazi	521,000	0.483	7,570	8,450	2,710	None	None
	(520,000, 522,000)	(0.458, 0.509)	(5,740, 9,400)	(5,860, 11,000)	(2,590, 2,830)		
Bugala (M) - Kiyindi	507,000	0.361	1.88	3,870	355	0.0000169	0
	(506,000, 508,000)	(0.318, 0.404)	(1.74, 2.01)	(1,810, 5,920)	(348, 362)	(-14.3, 14.3)	(-31,100, 31,100)
Bugala (M) - Wamala	521,000	0.51	13.1	323	186	0	0.00226
	(520,000, 521,000)	(0.491, 0.53)	(9.03, 17.1)	(-168, 814)	(179, 194)	(-162, 162)	(-3,980, 3,980)
Buwama - Kaazi	523,000	0.553	706	186	181	3.16	2.12
	(522,000, 524,000)	(0.357, 0.749)	(-764, 2,180)	(-101, 472)	(122, 239)	(-5,690, 5,690)	(-836, 841)
Buwama - Kiyindi	511,000	0.322	9,110	9,810	369	761	178
	(510,000, 512,000)	(0.287, 0.357)	(5,390, 12,800)	(4,390, 15,200)	(302, 436)	(-80,200, 81,700)	(-81,200, 81,500)
Buwama - Wamala	523,000	0.365	31.7	19.7	124	9.27	0
	(522,000, 524,000)	(-1,000, 1,000)	(22, 41.3)	(13.6, 25.8)	(107, 141)	(-578, 596)	(-344, 344)
Kaazi - Kiyindi	510,000	0.448	9,900	1,670	438	923	718
	(509,000, 511,000)	(0.325, 0.571)	(1,100, 18,700)	(749, 2,600)	(365, 510)	(-64,900, 66,800)	(610, 825)
Kaazi - Wamala	521,000	0.581	9,200	4,820	974	None	None
	(520,000, 522,000)	(0.515, 0.647)	(3,680, 14,700)	(2,430, 7,210)	(795, 1,150)		
Kiyindi - Wamala	510,000	0.454	134	40.3	181	0	0.00363
	(509,000, 511,000)	(0.391, 0.518)	(57.9, 209)	(25.4, 55.3)	(166, 197)	(-663, 663)	(-3.69, 3.7)

Table S6: Locality-specific (in LVB) putative sweeps based on H12 statistic.

Site	Count	Chr.	Putative Sweeps	Other sites[1]
Banda	44	2L	28.6 Mb; 36 Mb; 36.4 Mb; 36.9 Mb; 37.6 Mb; 38.1 Mb; 39.1 Mb; 42.2 Mb; 43.4 Mb; 43.8 Mb; 44.3 Mb; 44.9 Mb; 45.4 Mb	1 also found in BFS, GNS
		2R	4.2 Mb; 12.3 Mb; 18.3 Mb; 23.6 Mb; 29.4 Mb; 30.3 Mb; 33.7 Mb; 34.8 Mb; 35.8 Mb; 36.5 Mb; 44.1 Mb; 44.6 Mb; 49.7 Mb	1 also found in BFM, BFS, CMS, GNS, GWA; 1 also found in BFM, GWA; 5 also found in GWA
		3L	18.5 Mb; 21.6 Mb; 23.4 Mb; 23.9 Mb; 32.8 Mb	
		3R	2.6 Mb; 7.9 Mb; 29.2 Mb; 30.5 Mb; 31.3 Mb; 32.1 Mb; 33.2 Mb; 45.3 Mb; 46.4 Mb; 47 Mb	1 also found in GNS
		X	0.5 Mb; 2.1 Mb; 4.3 Mb	1 also found in AOM
Bugala (I)	24	2L	2.5 Mb; 5.5 Mb; 7.1 Mb; 19 Mb; 31.1 Mb; 43 Mb; 45.7 Mb	1 also found in AOM, BFM, BFS, CMS, GAS, GNS, UGS; 1 also found in AOM, UGS
		2R	6.7 Mb; 21.1 Mb; 24 Mb; 24.6 Mb; 35.6 Mb; 37.1 Mb; 38.6 Mb; 39 Mb; 55.9 Mb	1 also found in BFM, GWA; 2 also found in GWA
		3L	17.2 Mb; 29.5 Mb	
		3R	26 Mb; 35.8 Mb; 37.5 Mb	
		X	3.5 Mb; 5.7 Mb; 10.8 Mb	
Bukasa	112	2L	12.6 Mb; 13.6 Mb; 17.7 Mb; 20.1 Mb; 20.9 Mb; 21.6 Mb; 22.7 Mb; 23.6 Mb; 24.7 Mb; 25.4 Mb; 26.2 Mb; 26.9 Mb; 27.3 Mb; 27.8 Mb; 28.4 Mb; 29.1 Mb; 30.1 Mb; 31.5 Mb; 32.3 Mb; 33.3 Mb; 35.8 Mb; 39.4 Mb; 39.8 Mb; 40.6 Mb; 41.4 Mb; 43.1 Mb; 45.6 Mb; 48.1 Mb; 49.3 Mb	1 also found in AOM, BFM, BFS, CMS, GAS, GNS; 1 also found in BFM, GAS; 1 also found in BFS, GAS, GNS; 1 also found in BFS, GNS; 2 also found in CMS; 2 also found in GAS; 1 also found in GWA

		2R	1.3 Mb; 4.7 Mb; 5.3 Mb; 7.2 Mb; 7.6 Mb; 8 Mb; 9.7 Mb; 10.5 Mb; 12 Mb; 12.4 Mb; 13.5 Mb; 14 Mb; 15.7 Mb; 16.9 Mb; 17.5 Mb; 19.4 Mb; 22.9 Mb; 24.9 Mb; 25.8 Mb; 26.6 Mb; 29.9 Mb; 30.8 Mb; 32.4 Mb; 33.4 Mb; 35.5 Mb; 37.6 Mb; 43 Mb; 45.6 Mb; 47 Mb; 49.5 Mb; 54.8 Mb	1 also found in AOM, GAS, GWA; 2 also found in BFM; 1 also found in BFM, GWA; 3 also found in GAS, GWA; 6 also found in GWA
		3L	7.3 Mb; 11.6 Mb; 13.1 Mb; 15.6 Mb; 18.1 Mb; 19.1 Mb; 19.8 Mb; 20.6 Mb; 24.2 Mb; 25.2 Mb; 27.3 Mb; 28 Mb; 28.7 Mb; 29.7 Mb; 30.6 Mb; 33.7 Mb; 34.7 Mb; 35.3 Mb; 36.1 Mb; 38.7 Mb; 39.9 Mb; 40.3 Mb; 41.2 Mb	2 also found in BFM; 1 also found in GAS
		3R	5.1 Mb; 5.9 Mb; 7.2 Mb; 8.9 Mb; 12.7 Mb; 13.3 Mb; 14.1 Mb; 14.9 Mb; 15.9 Mb; 17.2 Mb; 22.3 Mb; 23.3 Mb; 23.8 Mb; 24.9 Mb; 26.8 Mb; 27.9 Mb; 31.4 Mb; 33 Mb; 35.9 Mb; 36.9 Mb	1 also found in GWA
		X	1.7 Mb; 2.8 Mb; 4.9 Mb; 6 Mb; 7 Mb; 11.5 Mb; 12.5 Mb; 13.6 Mb; 16.7 Mb	1 also found in BFM, GAS, GWA; 4 also found in GAS
Buwama	27	2L	14.9 Mb; 15.9 Mb; 25.1 Mb; 26.5 Mb; 31.6 Mb	1 also found in BFM, GAS; 1 also found in GWA
		2R	24.4 Mb; 39.5 Mb; 44.5 Mb; 46.3 Mb; 49.1 Mb; 53.7 Mb; 55.3 Mb	1 also found in AOM, BFM, CMS; 1 also found in BFS, CMS, GNS; 1 also found in CMS; 1 also found in GWA
		3L	2.4 Mb; 3.1 Mb; 3.6 Mb; 4.1 Mb; 10.6 Mb; 16.1 Mb; 21.7 Mb; 29.8 Mb	
		3R	18 Mb; 29.1 Mb; 35.5 Mb; 37.7 Mb; 38.4 Mb; 38.9 Mb; 40.6 Mb	1 also found in GNS

Kaazi	15	2L	8.5 Mb; 34.6 Mb	1 also found in AOM
		2R	8.3 Mb; 23 Mb	1 also found in AOM; 1 also found in GAS
		3L	3.5 Mb; 4.8 Mb; 8.6 Mb; 11.8 Mb; 13 Mb; 15.8 Mb; 25 Mb; 26.8 Mb	1 also found in BFM; 1 also found in BFM, GNS
		3R	14.7 Mb; 15.6 Mb; 46 Mb	1 also found in GNS
Kiyindi	40	2L	2 Mb; 10.6 Mb; 17.8 Mb; 22.1 Mb; 23.9 Mb; 26 Mb; 28.7 Mb; 29.9 Mb; 34.8 Mb	1 also found in AOM, BFM, BFS, CMS, GNS, UGS; 1 also found in BFS, GNS; 3 also found in GAS
		2R	19.1 Mb; 20.2 Mb; 25.9 Mb; 35.3 Mb; 36.6 Mb; 38.1 Mb; 40 Mb; 41.7 Mb; 42.4 Mb; 45.3 Mb; 48.2 Mb; 48.6 Mb; 50.1 Mb; 52.2 Mb; 53.6 Mb; 54.7 Mb; 55.1 Mb	1 also found in AOM; 1 also found in AOM, BFS, CMS, GNS, GWA; 1 also found in BFM; 2 also found in GWA
		3L	1.2 Mb; 8.9 Mb; 12.1 Mb; 12.6 Mb; 13.5 Mb; 14.8 Mb; 15.4 Mb; 16 Mb; 16.8 Mb; 19.7 Mb; 26.7 Mb	1 also found in BFM; 1 also found in GWA
		3R	38 Mb; 41.9 Mb; 48.3 Mb	
Nsadzi	47	2L	23.2 Mb; 27 Mb; 45.5 Mb	
		2R	1.6 Mb; 2.3 Mb; 3.2 Mb; 4 Mb; 8.8 Mb; 10.2 Mb; 13.2 Mb; 16.1 Mb; 20 Mb; 21.3 Mb; 24.7 Mb; 30.5 Mb; 34.2 Mb; 37.3 Mb; 41.2 Mb; 43.5 Mb; 52 Mb	1 also found in BFM, GAS; 1 also found in BFM, GWA; 1 also found in BFS, CMS, GNS; 1 also found in CMS; 2 also found in GWA
		3L	10.5 Mb; 11 Mb; 24.3 Mb; 35 Mb; 35.4 Mb; 36.8 Mb; 37.6 Mb	1 also found in GAS
		3R	3.8 Mb; 6 Mb; 7.4 Mb; 19.9 Mb; 20.5 Mb; 21.4 Mb; 23 Mb; 24.2 Mb; 27.7 Mb; 41.6 Mb; 42.2 Mb; 48.2 Mb; 49.8 Mb; 50.4 Mb	1 also found in BFS, GNS
		X	0.7 Mb; 2.3 Mb; 5.2 Mb; 7.7 Mb; 11.9 Mb; 17.9 Mb	1 also found in BFM, GAS, GWA; 1 also found in GAS

Sserinya	35	2L	22.2 Mb; 24.2 Mb; 25.7 Mb; 33.2 Mb; 34.9 Mb; 35.4 Mb; 40.2 Mb; 41.1 Mb; 45.1 Mb; 45.9 Mb; 46.8 Mb	1 also found in BFM, GNS; 1 also found in CMS, GAS; 1 also found in GAS, GNS
		2R	0.4 Mb; 7.7 Mb; 21.5 Mb; 30 Mb; 32 Mb; 36.1 Mb	3 also found in GWA
		3L	10.1 Mb; 10.9 Mb; 14.6 Mb; 34.5 Mb; 41.8 Mb	1 also found in BFS, CMS, GNS, GWA, UGS
		3R	1.9 Mb; 10 Mb; 15 Mb; 24.8 Mb; 26.2 Mb; 27 Mb; 29 Mb	1 also found in GAS
		X	5.8 Mb; 12.7 Mb; 13.1 Mb; 18.1 Mb; 18.8 Mb; 21.3 Mb	1 also found in BFM, CMS, GAS, GWA; 1 also found in BFM, GAS, GWA; 1 also found in CMS, GNS, GWA; 2 also found in GAS
Wamala	25	2L	13.4 Mb; 15.5 Mb; 17.1 Mb; 19.1 Mb; 20 Mb	2 also found in GAS
		2R	21.2 Mb; 22.2 Mb; 29.6 Mb; 38.8 Mb; 39.7 Mb; 47.6 Mb; 48.3 Mb; 48.9 Mb	2 also found in AOM; 2 also found in GWA
		3L	3.3 Mb; 7.6 Mb; 8.2 Mb	
		3R	5 Mb; 39.2 Mb; 43.2 Mb; 46.5 Mb; 47.5 Mb; 48.5 Mb; 50.5 Mb; 50.9 Mb; 51.8 Mb	

[1] Ag1000G site codes: AOM: Angola [*coluzzii*]; BFM: Burkina Faso [*coluzzii*]; BFS: Burkina Faso [*gambiae*]; CMS: Cameroon [*gambiae*]; GAS: Gabon [*gambiae*]; GNS: Guinea [*gambiae*]; GWA: Guinea-Bissau; UGS: Uganda [*gambiae*]

Table S7: Putative sweeps based on H12 statistic present on islands but rare or absent on LVB mainland.

Chr.	Region Start	Region End	Island Sites with Putative Sweep	Mainland Sites with Putative Sweep	Outlier Island Localities	Outlier Mainland Localities	Ag1000G Populations with Putative Sweep
2R	16,200,000	16,300,000	4 / 5	0 / 4	Banda; Bukasa; Nsadzi; Sserinya	None	Guinea-Bissau
2R	17,300,000	17,500,000	4 / 5	1 / 4	Banda; Bugala (I); Bukasa; Sserinya	Buwama	Guinea-Bissau
2R	21,000,000	21,100,000	5 / 5	1 / 4	Banda; Bugala (I); Bukasa; Nsadzi; Sserinya	Buwama	None
2R	40,400,000	40,800,000	4 / 5	1 / 4	Bugala (I); Bukasa; Nsadzi; Sserinya	Wamala	Burkina Faso [*gambiae*], Cameroon [*gambiae*], Gabon [*gambiae*], Guinea-Bissau
2R	41,100,000	41,200,000	4 / 5	1 / 4	Banda; Bukasa; Nsadzi; Sserinya	Wamala	Cameroon [*gambiae*]
2R	55,800,000	55,900,000	4 / 5	1 / 4	Banda; Bugala (I); Bukasa; Sserinya	Kiyindi	Angola [*coluzzii*]
2L	7,700,000	7,800,000	4 / 5	1 / 4	Banda; Bugala (I); Bukasa; Nsadzi	Buwama	Guinea-Bissau, Uganda [*gambiae*]
2L	8,100,000	8,200,000	4 / 5	0 / 4	Banda; Bugala (I); Nsadzi; Sserinya	None	None
2L	42,400,000	42,500,000	4 / 5	0 / 4	Banda; Bugala (I); Nsadzi; Sserinya	None	None
2L	43,500,000	43,600,000	5 / 5	1 / 4	Banda; Bugala (I); Bukasa; Nsadzi; Sserinya	Buwama	None
2L	49,000,000	49,100,000	4 / 5	1 / 4	Banda; Bugala (I); Bukasa; Sserinya	Wamala	None

3R	26,600,000	26,700,000	5 / 5	0 / 4	Banda; Bugala (I); Bukasa; Nsadzi; Sserinya	None	None
3R	36,700,000	36,800,000	4 / 5	0 / 4	Banda; Bugala (I); Bukasa; Nsadzi	None	None
3R	44,200,000	44,300,000	4 / 5	1 / 4	Banda; Bukasa; Nsadzi; Sserinya	Kiyindi	Angola [*coluzzii*]
3R	46,200,000	46,300,000	4 / 5	0 / 4	Banda; Bukasa; Nsadzi; Sserinya	None	None
X	6,600,000	7,000,000	4 / 5	0 / 4	Banda; Bugala (I); Bukasa; Nsadzi; Sserinya	None	Burkina Faso [*coluzzii*], Gabon [*gambiae*], Guinea-Bissau
X	8,100,000	10,700,000	4 / 5	0 / 4	Banda; Bugala (I); Bukasa; Nsadzi; Sserinya	Kiyindi	Burkina Faso [*coluzzii*], Burkina Faso [*gambiae*], Gabon [*gambiae*]
X	11,300,000	11,800,000	5 / 5	0 / 4	Banda; Bugala (I); Bukasa; Nsadzi; Sserinya	None	Gabon [*gambiae*]
X	12,900,000	13,000,000	4 / 5	0 / 4	Banda; Bugala (I); Bukasa; Sserinya	None	Gabon [*gambiae*]
X	14,300,000	14,400,000	5 / 5	1 / 4	Banda; Bugala (I); Bukasa; Nsadzi; Sserinya	Kaazi	Gabon [*gambiae*]
X	16,200,000	16,300,000	4 / 5	1 / 4	Banda; Bugala (I); Bukasa; Sserinya	Kaazi	Burkina Faso [*coluzzii*], Gabon [*gambiae*]

Table S8: Putative sweeps based on H12 statistic present on LVB mainland but rare or absent on islands.

Chr.	Region Start	Region End	Island Sites with Putative Sweep	Mainland Sites with Putative Sweep	Outlier Island Localities	Outlier Mainland Localities	Ag1000G Populations with Putative Sweep
2R	27,600,000	27,700,000	1 / 5	3 / 4	Nsadzi	Buwama; Kiyindi; Wamala	None
2R	38,000,000	38,100,000	1 / 5	3 / 4	Bugala (I)	Buwama; Kiyindi; Wamala	None
2R	42,700,000	42,800,000	0 / 5	3 / 4	None	Buwama; Kiyindi; Wamala	None
2R	45,400,000	45,500,000	1 / 5	3 / 4	Sserinya	Buwama; Kiyindi; Wamala	None
2R	46,800,000	46,900,000	1 / 5	3 / 4	Banda	Buwama; Kiyindi; Wamala	Cameroon [*gambiae*]
2R	48,000,000	48,100,000	1 / 5	3 / 4	Bukasa	Buwama; Kaazi; Wamala	Angola [*coluzzii*], Cameroon [*gambiae*]
2R	48,800,000	48,900,000	1 / 5	3 / 4	Nsadzi	Buwama; Kaazi; Wamala	None
2R	50,900,000	51,000,000	1 / 5	3 / 4	Bukasa	Kaazi; Kiyindi; Wamala	Burkina Faso [*gambiae*], Guinea [*gambiae*]
2R	51,500,000	51,600,000	0 / 5	3 / 4	None	Kaazi; Kiyindi; Wamala	None
2R	57,500,000	57,600,000	1 / 5	3 / 4	Banda	Buwama; Kaazi; Kiyindi	Angola [*coluzzii*], Guinea-Bissau
2L	2,900,000	3,000,000	1 / 5	4 / 4	Sserinya	Buwama; Kaazi; Kiyindi; Wamala	Angola [*coluzzii*], Burkina Faso [*coluzzii*], Burkina Faso [*gambiae*], Cameroon [*gambiae*], Gabon [*gambiae*], Guinea [*gambiae*], Uganda [*gambiae*]
2L	4,200,000	4,300,000	1 / 5	4 / 4	Bugala (I)	Buwama; Kaazi; Kiyindi; Wamala	Angola [*coluzzii*], Cameroon [*gambiae*], Gabon [*gambiae*], Uganda [*gambiae*]

2L	5,700,000	5,800,000	1 / 5	3 / 4	Bugala (I)	Buwama; Kaazi; Kiyindi	Angola [*coluzzii*], Guinea [*gambiae*], Uganda [*gambiae*]
2L	6,200,000	6,300,000	1 / 5	3 / 4	Bugala (I)	Kaazi; Kiyindi; Wamala	Uganda [*gambiae*]
2L	6,600,000	6,800,000	1 / 5	3 / 4	Bugala (I)	Kaazi; Kiyindi; Wamala	Angola [*coluzzii*], Cameroon [*gambiae*], Gabon [*gambiae*], Guinea [*gambiae*], Uganda [*gambiae*]
2L	10,000,000	10,100,000	1 / 5	3 / 4	Sserinya	Kaazi; Kiyindi; Wamala	None
2L	10,800,000	10,900,000	0 / 5	3 / 4	None	Kaazi; Kiyindi; Wamala	None
2L	11,300,000	11,400,000	1 / 5	3 / 4	Bugala (I)	Kaazi; Kiyindi; Wamala	Guinea-Bissau
2L	12,000,000	12,100,000	1 / 5	3 / 4	Bugala (I)	Kaazi; Kiyindi; Wamala	None
2L	12,400,000	13,000,000	0 / 5	3 / 4	Bukasa	Buwama; Kaazi; Kiyindi; Wamala	None
2L	14,500,000	14,900,000	1 / 5	3 / 4	Sserinya	Buwama; Kiyindi; Wamala	Gabon [*gambiae*], Uganda [*gambiae*]
2L	16,000,000	16,300,000	1 / 5	3 / 4	Bukasa	Buwama; Kaazi; Wamala	Gabon [*gambiae*]
2L	16,600,000	16,700,000	1 / 5	4 / 4	Bugala (I)	Buwama; Kaazi; Kiyindi; Wamala	None
2L	18,700,000	18,800,000	1 / 5	3 / 4	Nsadzi	Kaazi; Kiyindi; Wamala	None
2L	33,600,000	33,700,000	1 / 5	3 / 4	Bugala (I)	Buwama; Kaazi; Kiyindi	Angola [*coluzzii*]
2L	34,400,000	34,500,000	1 / 5	3 / 4	Sserinya	Buwama; Kaazi; Wamala	None

3R	28,500,000	28,700,000	1 / 5	4 / 4	Sserinya	Buwama; Kaazi; Kiyindi; Wamala	Burkina Faso [*coluzzii*], Burkina Faso [*gambiae*], Cameroon [*gambiae*], Gabon [*gambiae*], Guinea [*gambiae*], Uganda [*gambiae*]
3R	36,500,000	36,900,000	0 / 5	3 / 4	Nsadzi	Buwama; Kiyindi; Wamala	None
3R	43,000,000	43,100,000	0 / 5	3 / 4	None	Buwama; Kiyindi; Wamala	None
3R	43,700,000	44,100,000	0 / 5	3 / 4	Nsadzi	Buwama; Kiyindi; Wamala	Angola [*coluzzii*], Burkina Faso [*gambiae*], Guinea [*gambiae*], Uganda [*gambiae*]
3R	48,800,000	48,900,000	0 / 5	3 / 4	None	Buwama; Kiyindi; Wamala	None
3R	50,000,000	50,100,000	1 / 5	3 / 4	Sserinya	Kaazi; Kiyindi; Wamala	None
3L	7,000,000	7,100,000	1 / 5	4 / 4	Sserinya	Buwama; Kaazi; Kiyindi; Wamala	None
3L	11,500,000	11,600,000	1 / 5	3 / 4	Sserinya	Buwama; Kiyindi; Wamala	Burkina Faso [*coluzzii*]
3L	12,200,000	12,300,000	0 / 5	3 / 4	None	Kaazi; Kiyindi; Wamala	None
3L	13,400,000	13,500,000	0 / 5	3 / 4	None	Kaazi; Kiyindi; Wamala	None
3L	16,300,000	16,400,000	1 / 5	3 / 4	Sserinya	Buwama; Kiyindi; Wamala	Uganda [*gambiae*]

Table S9: Signatures of selective sweeps on known insecticide genes by site based on H12 statistic.

Chr.	Location	Insecticide Gene	Island Sites with Putative Sweep	Mainland Sites with Putative Sweep	Outlier Island Localities	Outlier Mainland Localities	Ag1000G Populations with Putative Sweep
2R	28,497,407	Cyp6p	5 / 5	4 / 4	Banda; Bugala (I); Bukasa; Nsadzi; Sserinya	Buwama; Kaazi; Kiyindi; Wamala	Angola [*coluzzii*], Burkina Faso [*coluzzii*], Burkina Faso [*gambiae*], Cameroon [*gambiae*], Guinea [*gambiae*], Uganda [*gambiae*]
3R	28,598,038	Gste	1 / 5	4 / 4	Sserinya	Buwama; Kaazi; Kiyindi; Wamala	Burkina Faso [*coluzzii*], Burkina Faso [*gambiae*], Cameroon [*gambiae*], Gabon [*gambiae*], Guinea [*gambiae*], Uganda [*gambiae*]
X	15,241,718	Cyp9k1	3 / 5	4 / 4	Banda; Bukasa; Sserinya	Buwama; Kaazi; Kiyindi; Wamala	Burkina Faso [*coluzzii*], Burkina Faso [*gambiae*], Gabon [*gambiae*], Guinea [*gambiae*]

Table S10: Software and versions used for major parts of analysis.

Software	Version	Citation
ea-utils	-	Aronesty (2011)
BWA	0.7.16a	Li & Durbin (2009)
GATK	3.8	DePristo et al. (2011)
PLINK	1.90b4.6	Chang et al. (2015), Purcell et al. (2007)
SHAPEIT2	2.837	Delaneau et al. (2013)
SAMtools/BCFtools	1.5	Li (2011), Li et al. (2009)
ADMIXTURE	1.3.0	Alexander et al. (2009)
CLUMPAK	-	Kopelman et al. (2015)
VCFtools	0.1.15	Danecek et al. (2011)
$\delta a \delta i$ (python package)	1.7.0	Coffman et al. (2016), Gutenkunst et al. (2009)
Stairway plot - Jpopgen	2-beta	Liu & Fu (2015)
selscan	1.2.0a	Szpiech & Hernandez (2014)
adegenet (R package)	2.1.0	Jombart & Ahmed (2011)
ape (R package)	5.0	Paradis et al. (2004)
RColorBrewer (R package)	1.1-2	Neuwirth (2014)
dendextend (R package)	1.6.0	Galili (2015)
rehh (R package)	2.0.2	Gautier & Vitalis (2012)
eigensoft	7.2.1	Patterson et al. (2006), Price et al. (2006)
GNU parallel	20170422	Tange (2011)
tabix	1.5	Li (2011)
bedtools	2.26.0	Quinlan & Hall (2010)

Table S11: Genomic coordinates of heterochromatic and inverted regions.

Chromosome arm	Start	End	Information
2L	20,524,058	42,165,532	2La inversion Sharakhova et al. (2007)
2R	18,575,300	26,767,588	2Rb inversion Sharakhova et al. (2007)
2L	1	2,431,617	Heterochromatic region Sharakhova et al. (2007)
2L	5,078,962	5,788,875	Heterochromatic region Sharakhova et al. (2007)
2R	58,984,778	61,545,105	Heterochromatic region Sharakhova et al. (2007)
3L	1	1,815,119	Heterochromatic region Sharakhova et al. (2007)
3L	4,264,713	5,031,692	Heterochromatic region Sharakhova et al. (2007)
3R	38,988,757	41,860,198	Heterochromatic region Sharakhova et al. (2007)
3R	52,161,877	53,200,684	Heterochromatic region Sharakhova et al. (2007)
X	20,009,764	24,393,108	Heterochromatic region Sharakhova et al. (2007)

Figures

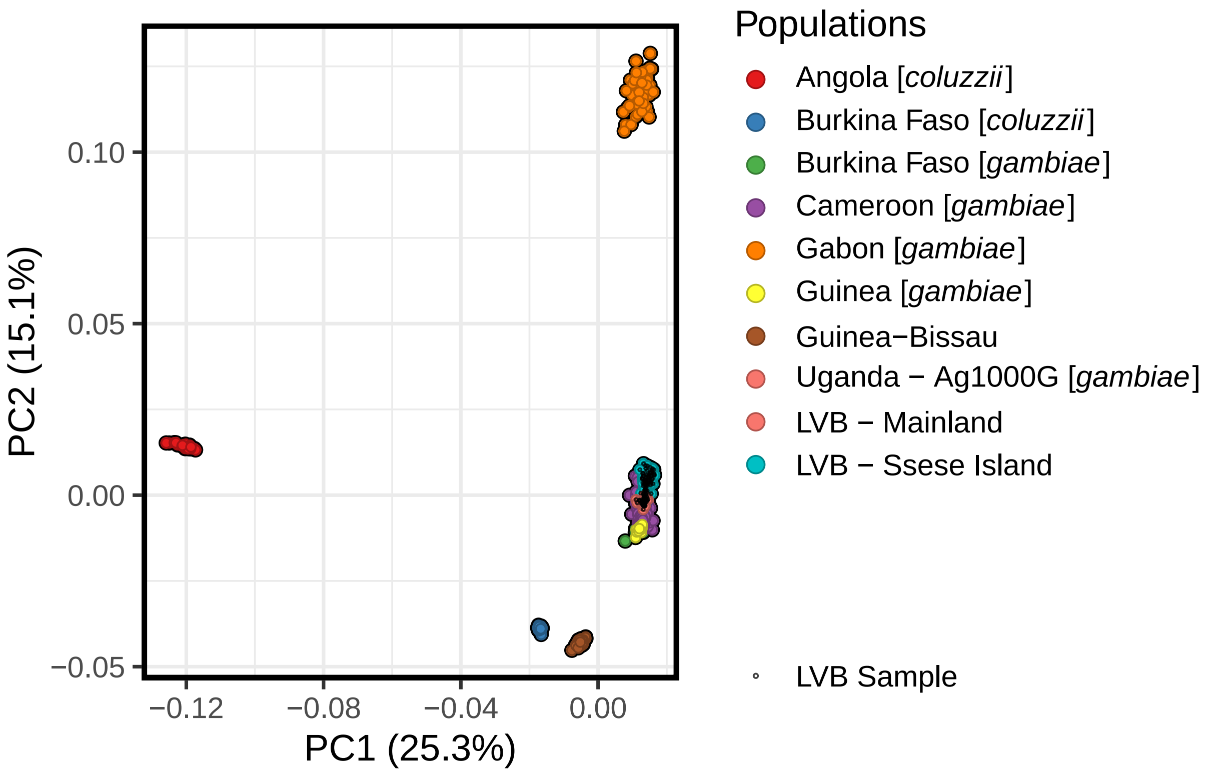

Figure S1: PCA plot of study individuals and *A. gambiae* and *A. coluzzii* individuals from reference Ag1000G populations, showing the first and second components. The outlier Kenyan population is not included for ease of visualization; this population exhibits inbreeding likely due to intensive insecticide use (Miles et al., 2017). Analysis is based on chromosome 3 to avoid the well-known inversions on chromosome 2 and the X-chromosome.

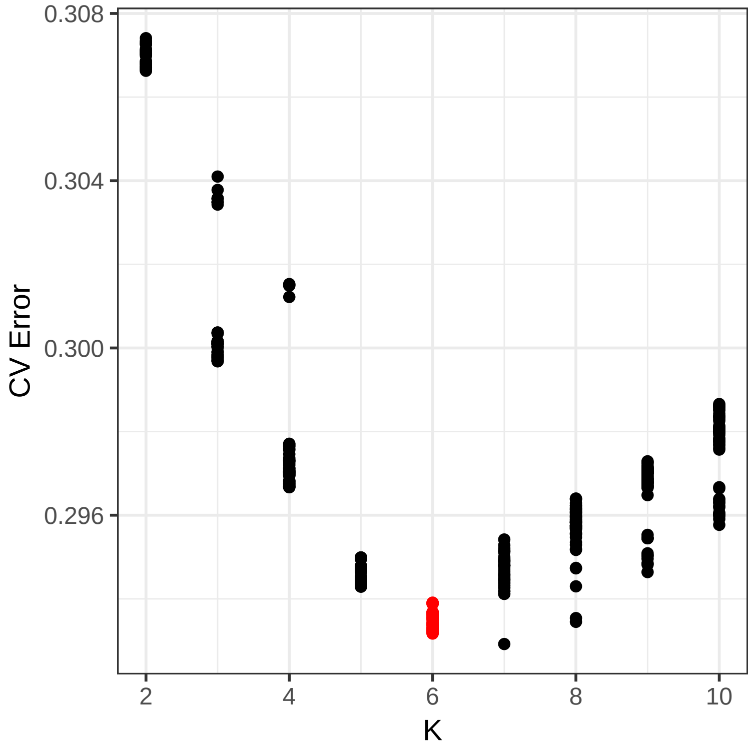

Figure S2: ADMIXTURE cross-validation error.
Cross-validation error for range of k values for ADMIXTURE analysis of Lake Victoria Basin individuals and *A. gambiae* and *A. coluzzii* Ag1000G reference populations.

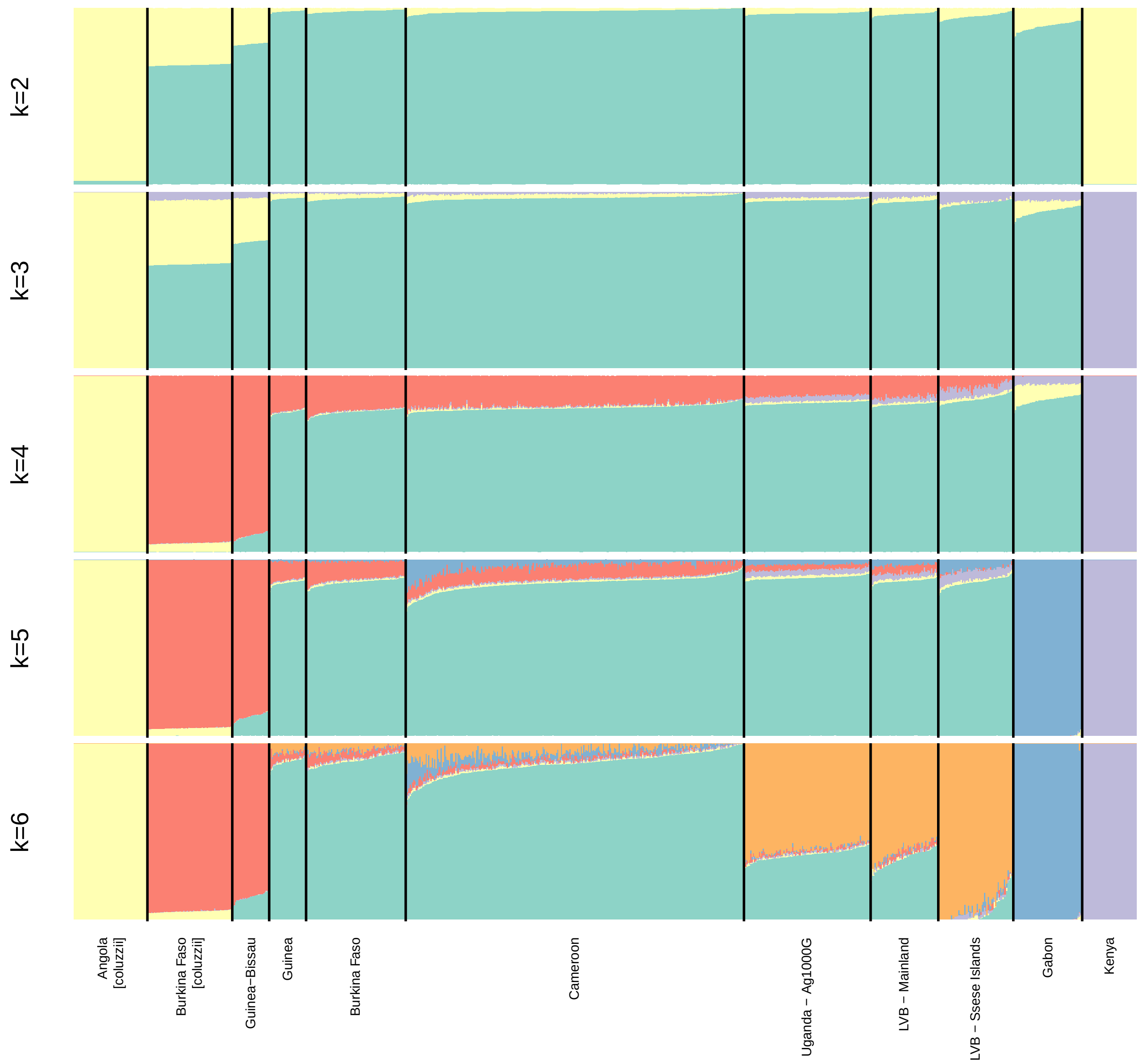

Figure S3: (Caption on next page.)

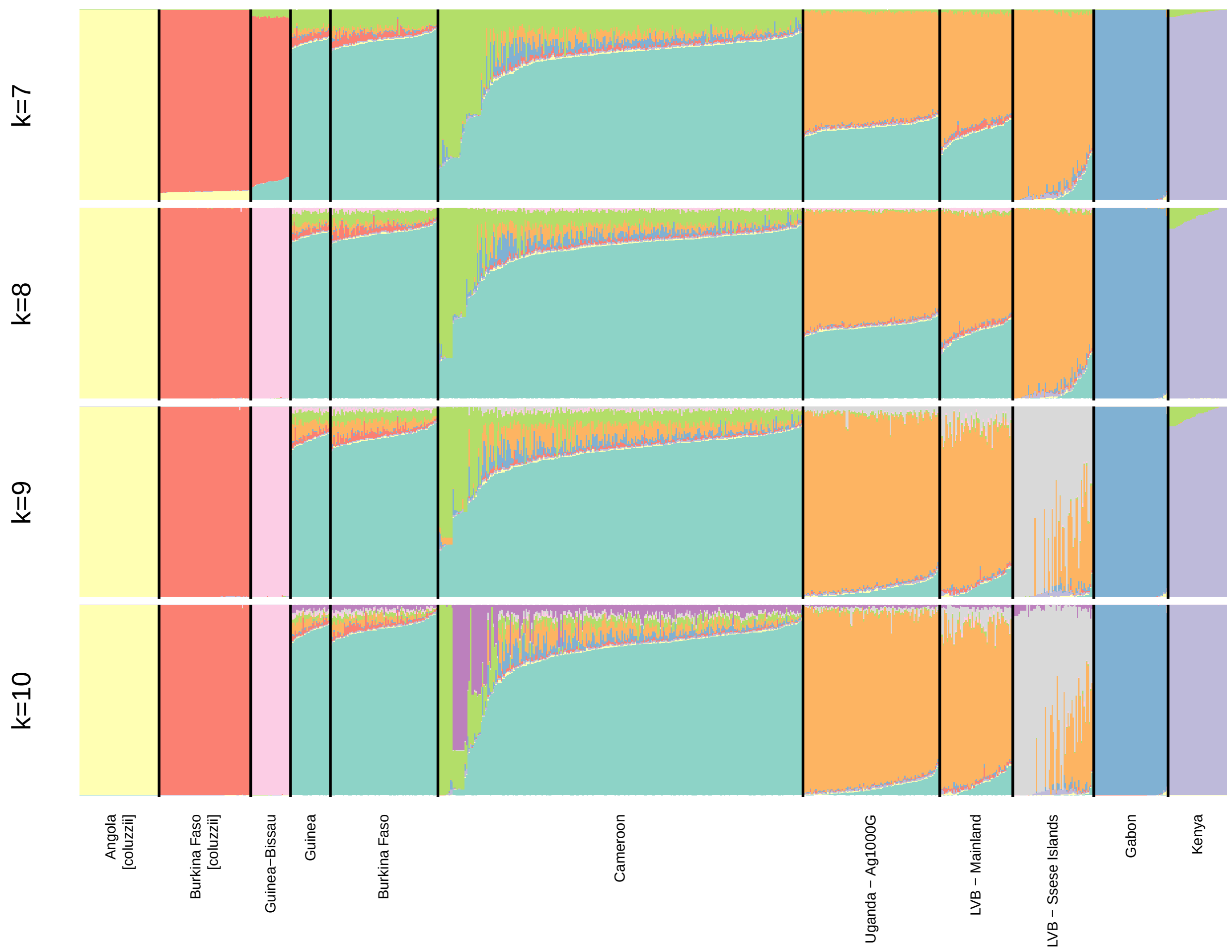

Figure S3: ADMIXTURE-inferred ancestry.

Ancestry of individuals in Lake Victoria Basin and of Ag1000G reference populations as inferred by ADMIXTURE clustering method. Samples are *A. gambiae* unless noted, and analysis is based on chromosome 3 to avoid the well-known inversions on chromosome 2 and the X-chromosome. Using $k = 6$ clusters minimizes cross validation error (Fig. S2).

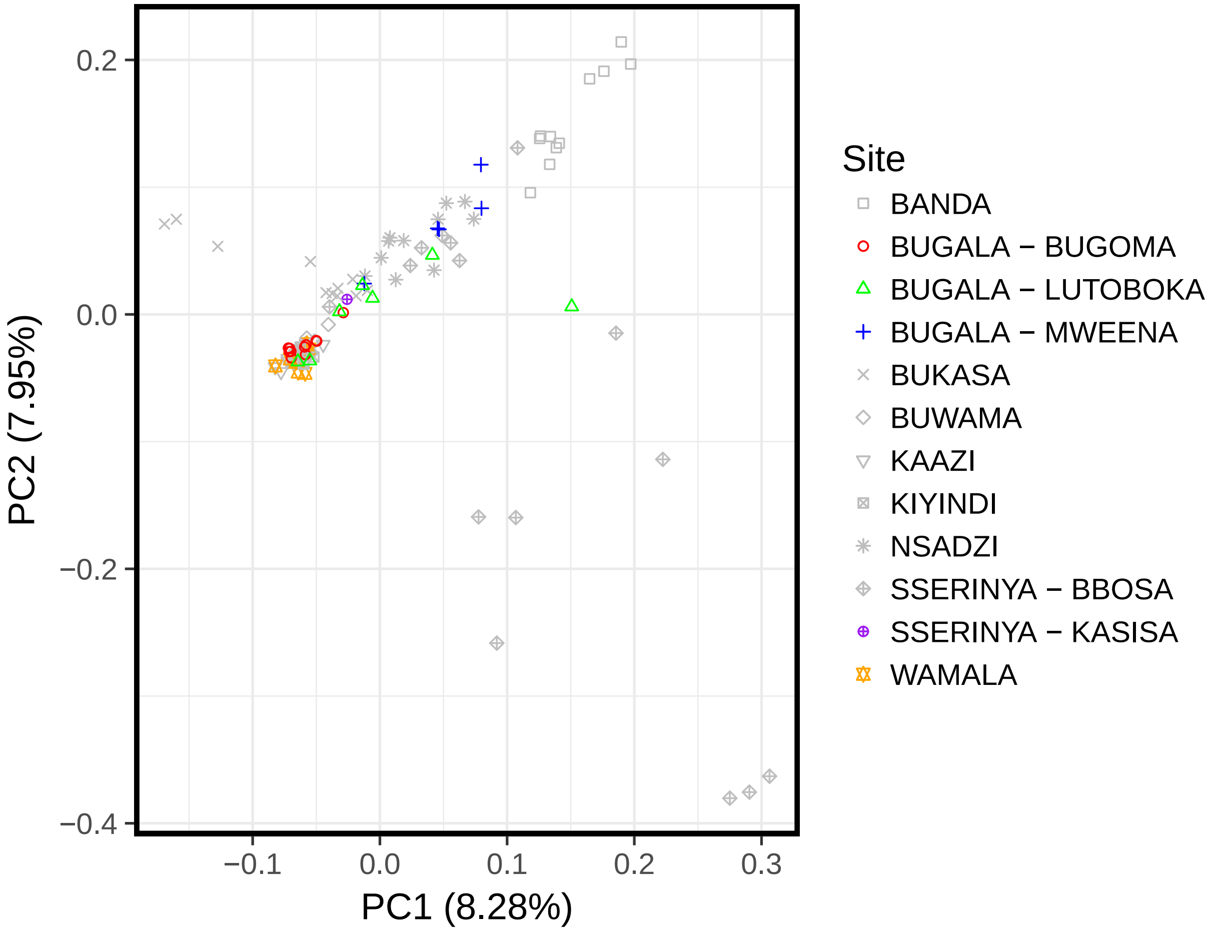

Figure S4: PCA showing Bugala subdivision.
PCA colored by sampling locations. Based on this analysis, individuals from Bugala were split into mainland- and island-like subpopulations. Samples from Sserinya Island, though sampled from two localities, were not split.

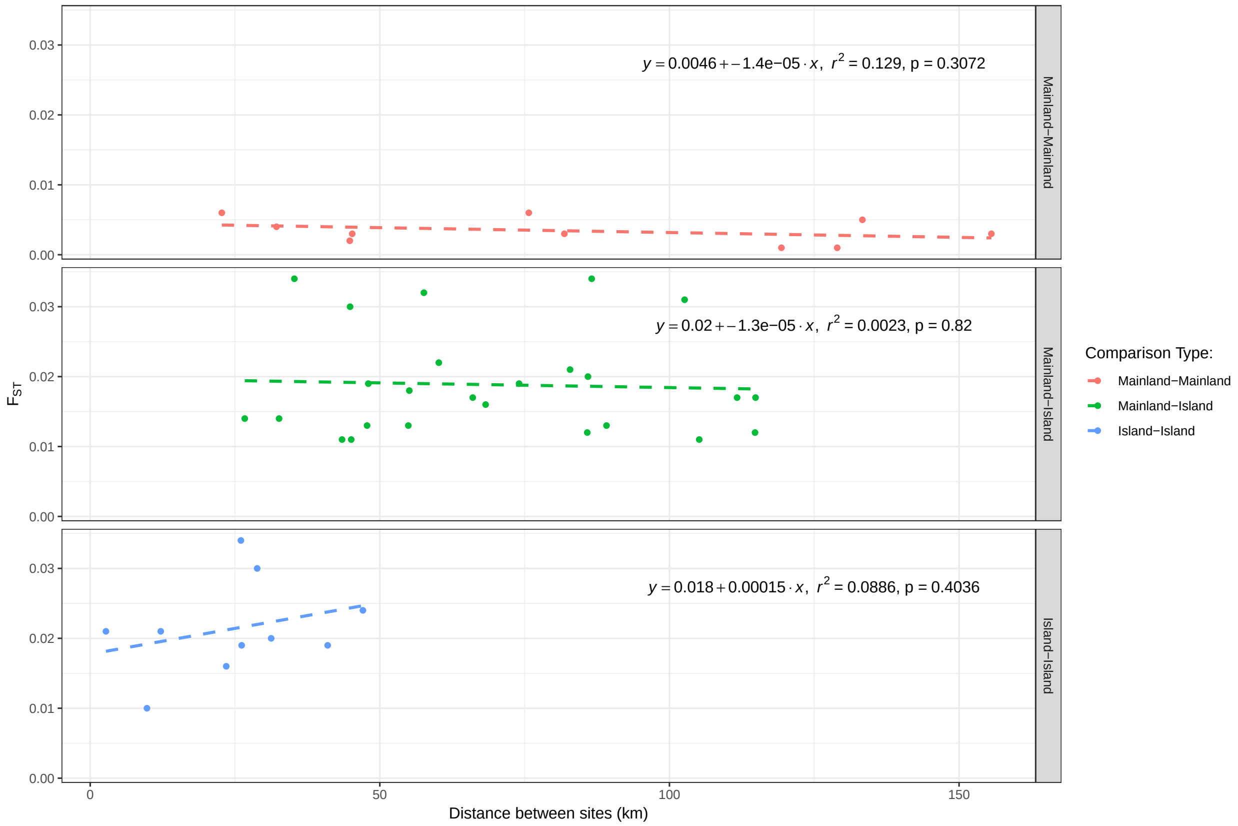

Figure S5: Correlations between genetic distance (F_{ST}) and geographic distance between localities within the LVB. The p-values are for the test that the slope is significantly different from zero.

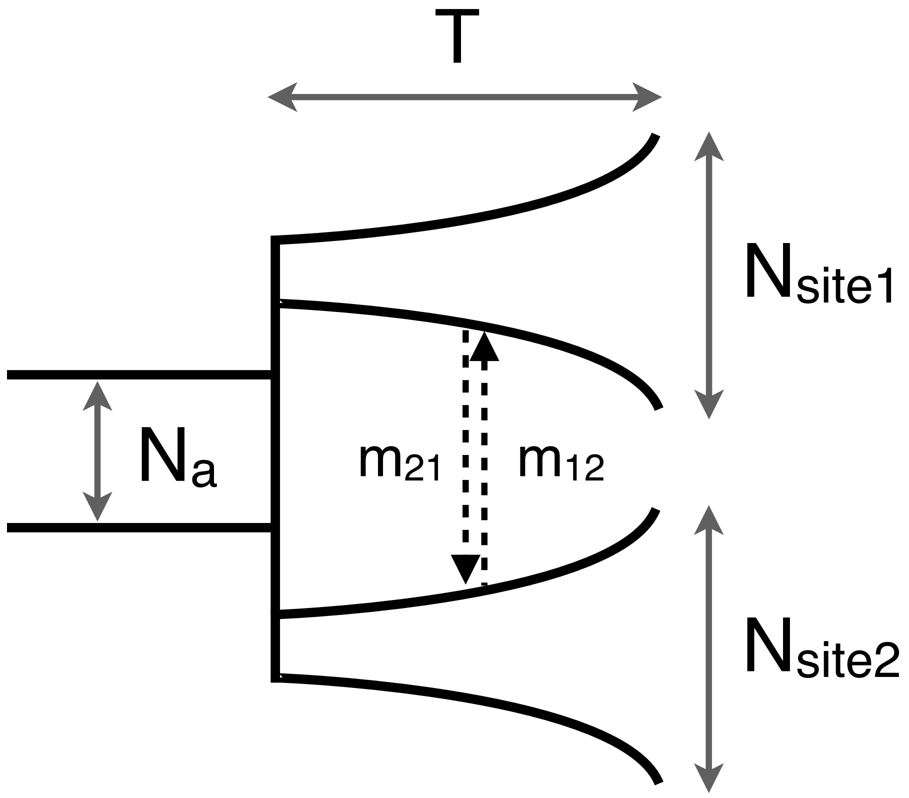

Figure S6: IM model schematics.
Schematic of model fit to data with δaδi for population history inference between all pairs of sampled sites using IM model.

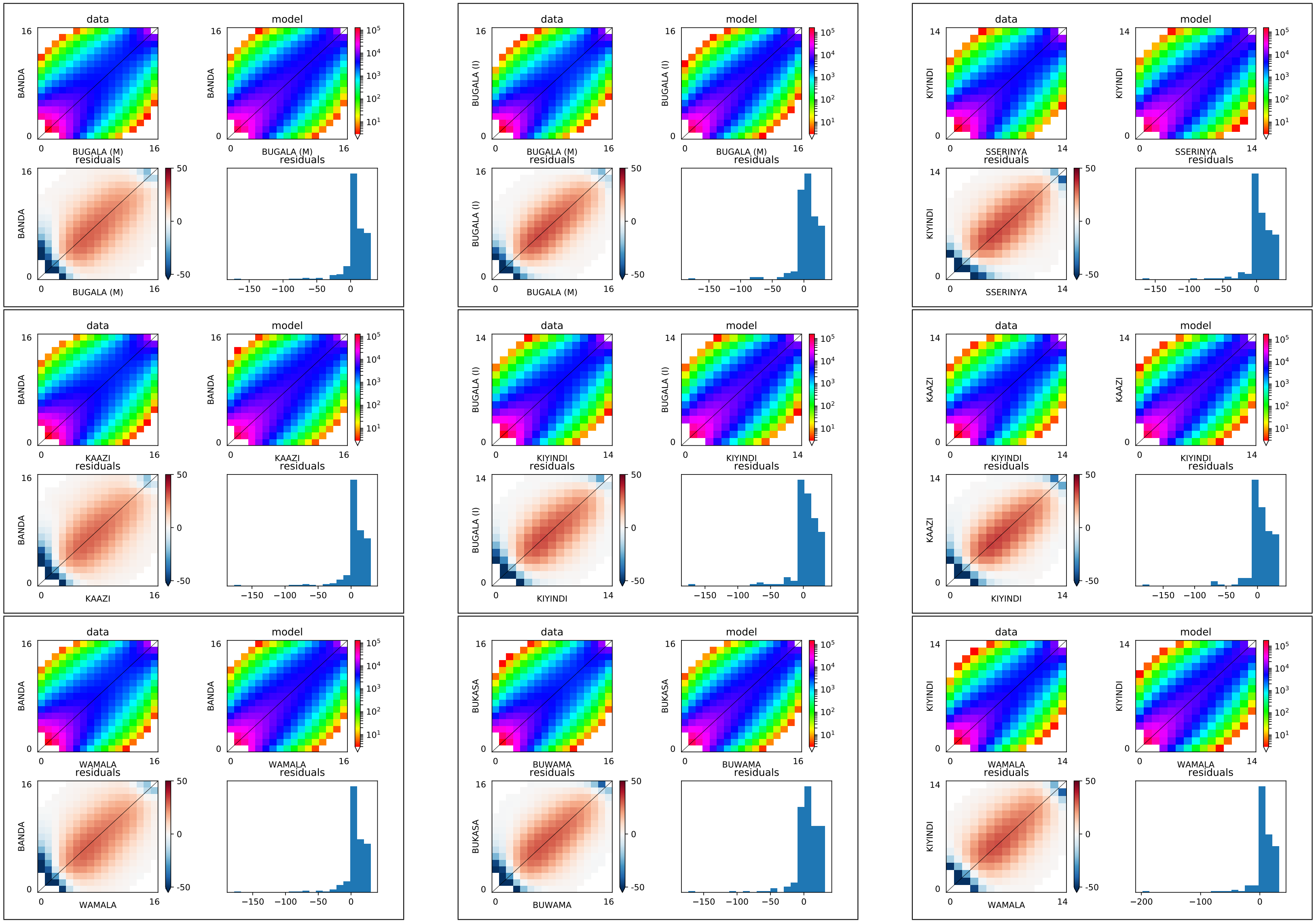

Figure S7: (Caption on next page.)

Figure S7: Two population δaδi optimization results.
Comparison between best fitting model and data frequency spectra for two population δaδi inference. Of the pairwise comparisons for which the best model included migration, a randomly selected set of nine are shown here. Two-dimensional frequency spectra are plotted as logarithmic colormaps for the data (upper left) and model (upper right), and the bottom row plots show the residuals between model and data. Positive residuals in red indicate the model predicts too many SNPs in that entry while negative residuals in blue indicate the model predicts too few.

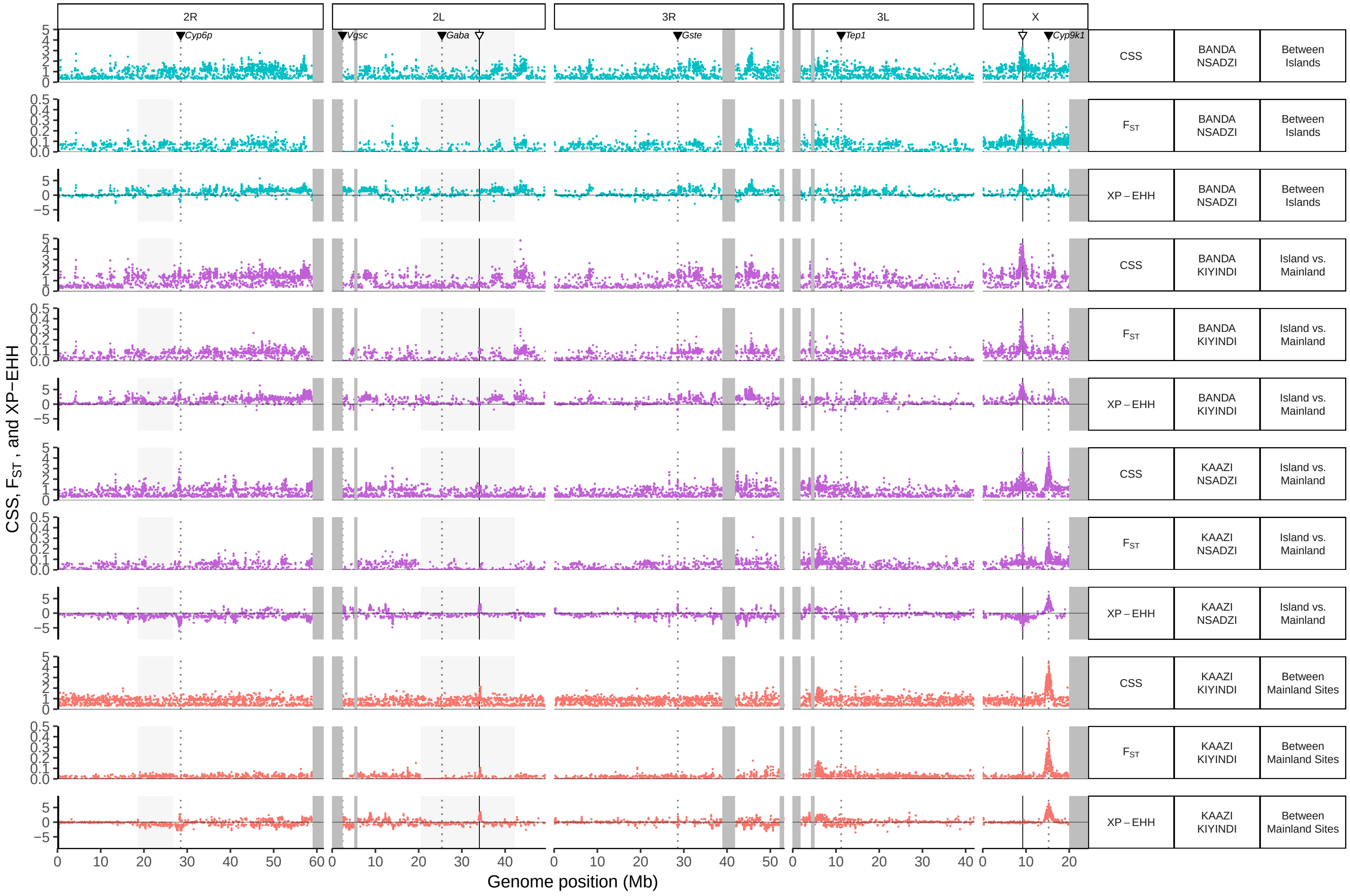

Figure S8: F_{ST}, XP-EHH, and Composite Selection Score (CSS) across genome.
F_{ST}, XP-EHH, and CSS averaged in windows of size 10 kb plotted across genome for pairwise comparisons of island and mainland localities. Shaded regions indicate inversions or heterochromatic regions (excluded from analysis) and dotted lines indicate known insecticide genes (and *Tep1*, a gene involved in resistance to *Plasmodium* infection) while solid lines indicate the two putative sweeps identified in the present study. Only several exemplar pairs of populations shown.

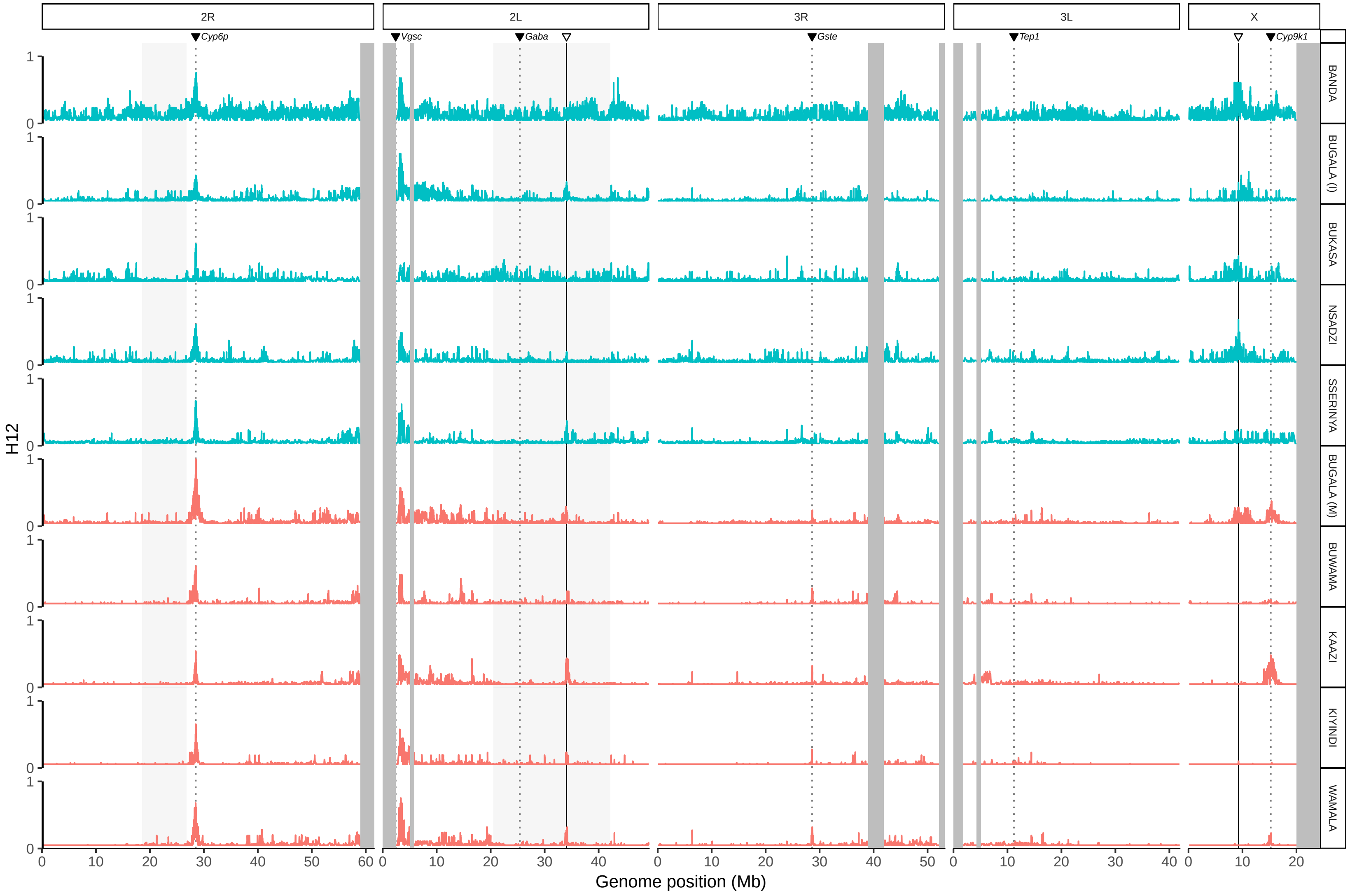

Figure S9: H12 across genome.

Values of H12, a measure of haplotype homozygosity, plotted across genome. Shaded regions indicate inversions or heterochromatic regions (excluded from analysis) and dotted lines indicate known insecticide genes (and *Tep1*, a gene involved in resistance to *Plasmodium* infection) while solid lines indicate the two putative sweeps identified in the present study.

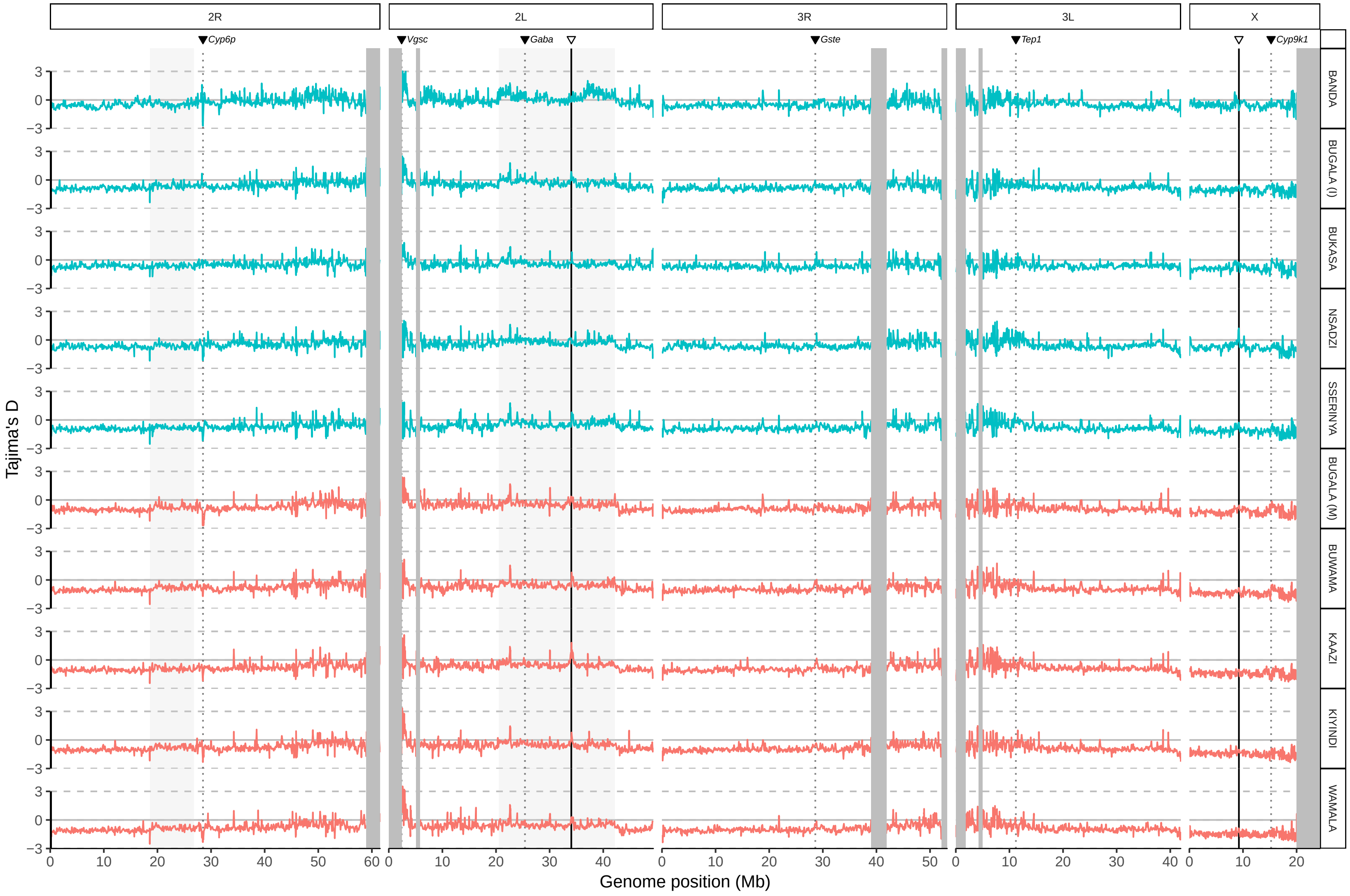

Figure S10: Tajima's D across genome.

Tajima's D plotted across genome. Shaded regions indicate inversions or heterochromatic regions (excluded from analysis) and dotted lines indicate known insecticide genes (and *Tep1*, a gene involved in resistance to *Plasmodium* infection) while solid lines indicate the two putative sweeps identified in the present study.

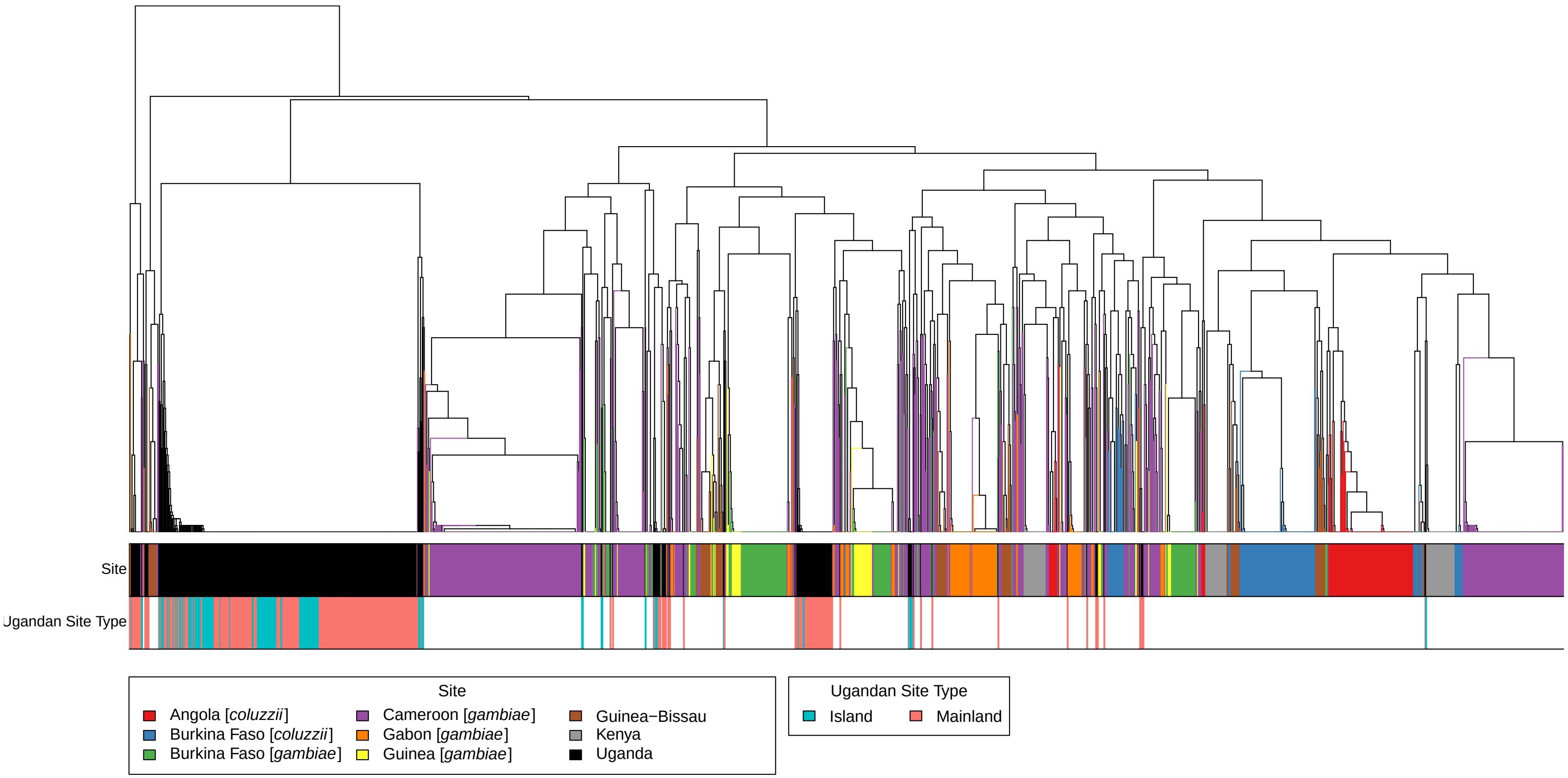

Figure S11: Tree for sweep on *Cyp6p* gene cluster on chromosome arm 2R.

Distance-based tree of haplotypes near sweep at *Cyp6p* gene cluster on chromosome arm 2R. Region shown is 10 kb up- and downstream of sweep target, centered at chr2R:28,501,972 (the approximate location of the peaks in pairwise statistics). Top color bar indicates locality, with all Ugandan individuals, from both the Ag1000G reference population and the LVB, in black. The bottom color bar differentiates the Ugandan individuals into mainland (red) and island (blue) individuals.

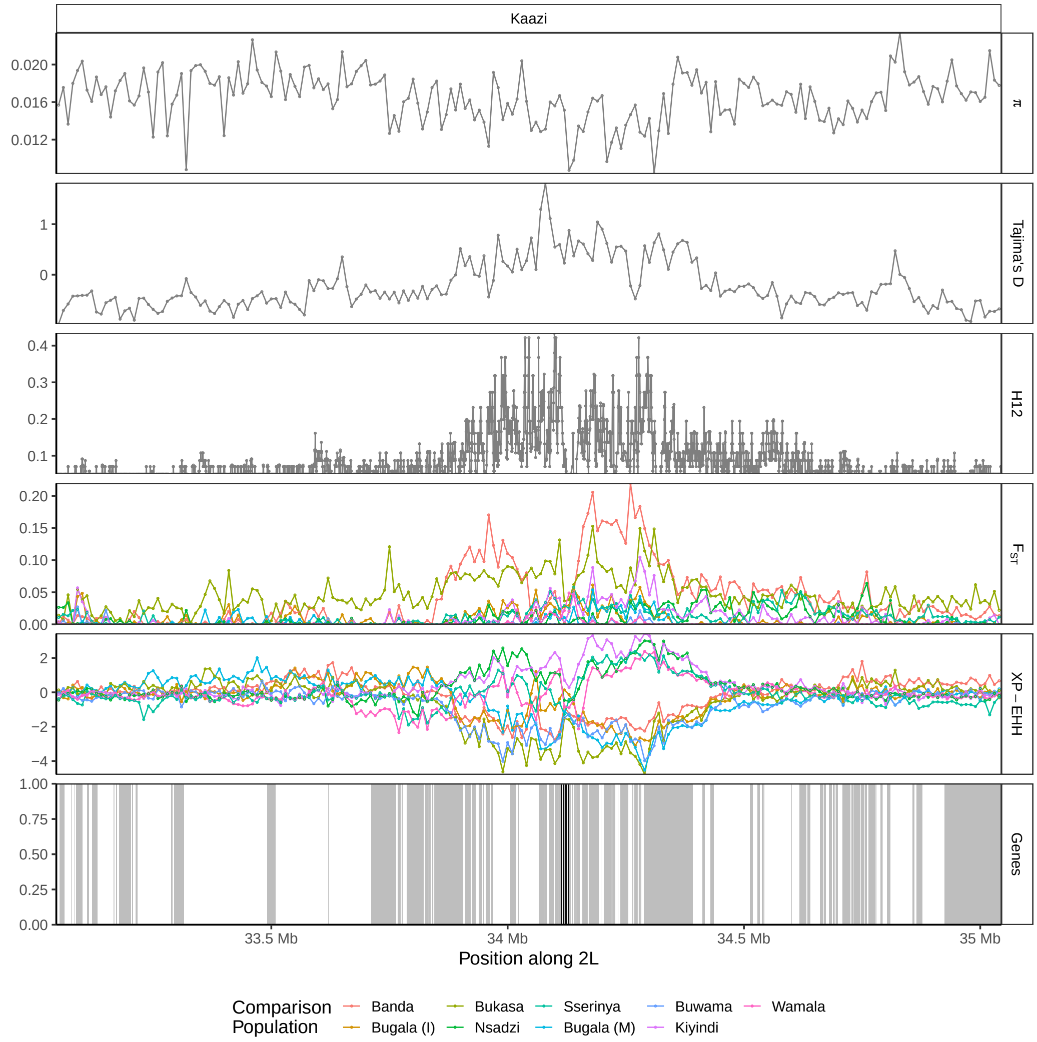

Figure S12: Selective sweep signal on chromosome 2L.

Population genetic statistics plotted near putative sweep on chromosome 2L. Focus population for all pairwise F_{ST} and XP-EHH comparisons is mainland site Kaazi, chosen to maximize peak height in these statistics. Region shown is 1 Mb up- and downstream of sweep target, centered at chr2L:34,044,820. Several genes involved in chorion formation (AGAP006549, AGAP006550, AGAP006551, AGAP006553, AGAP006554, AGAP006555 and AGAP006556) are highlighted with black vertical lines, while other genes are indicated with gray vertical lines.

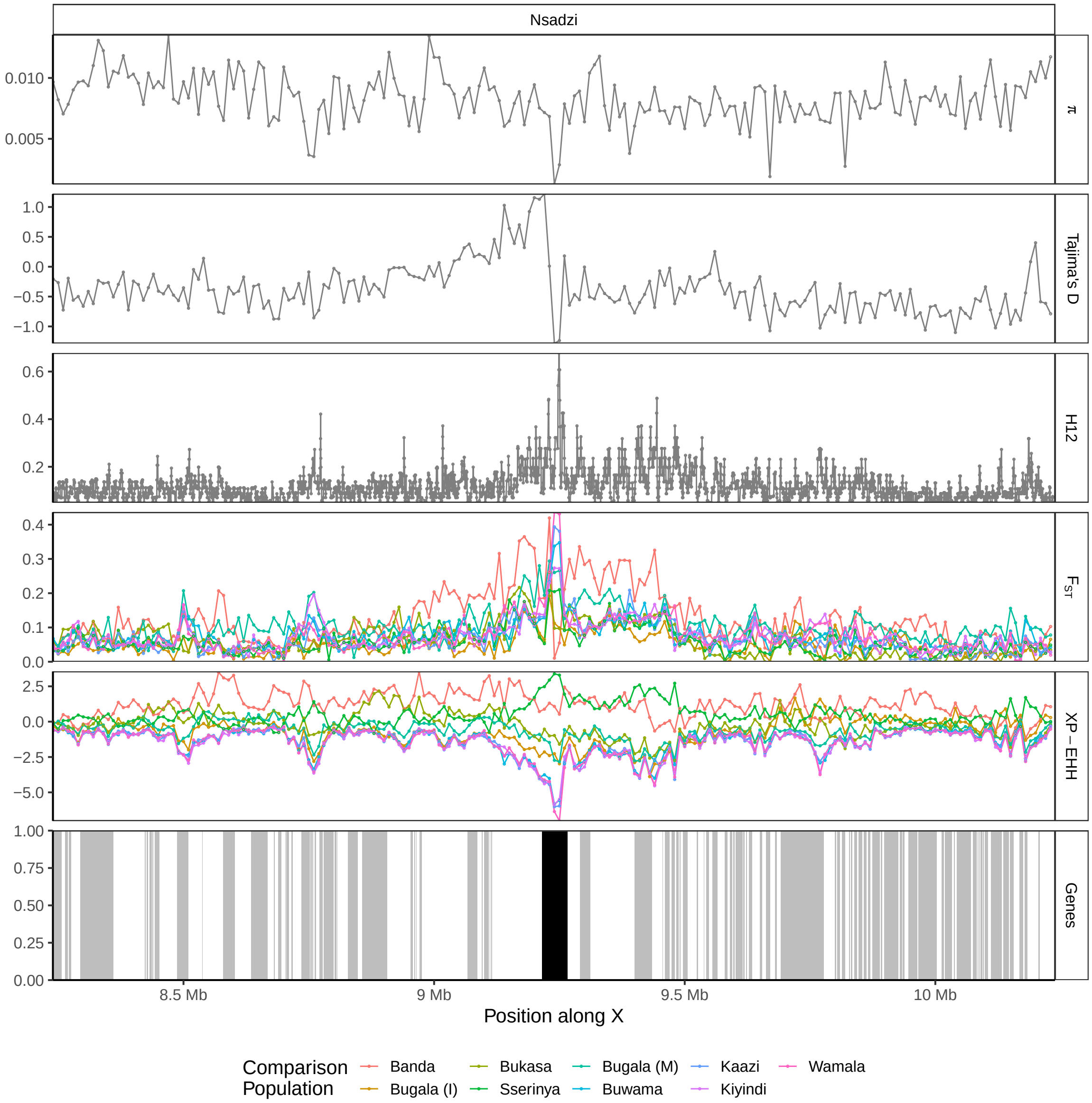

Figure S13: (Caption on next page.)

Figure S13: Selective sweep signal on X-chromosome near *rdgA* ortholog. Population genetic statistics plotted near putative sweep on X-chromosome. Focus population for all pairwise F_{ST} and XP-EHH comparisons is island site Nsadzi, chosen to maximize peak height in these statistics. Region shown is 1 Mb up- and downstream of sweep target, centered at chrX:9,238,942 (the approximate peak in pairwise statistics). The gene eye-specific diacylglycerol kinase (AGAP000519, chrX:9,215,505-9,266,532) is highlighted with a black vertical line, while other genes are indicated with gray vertical lines.

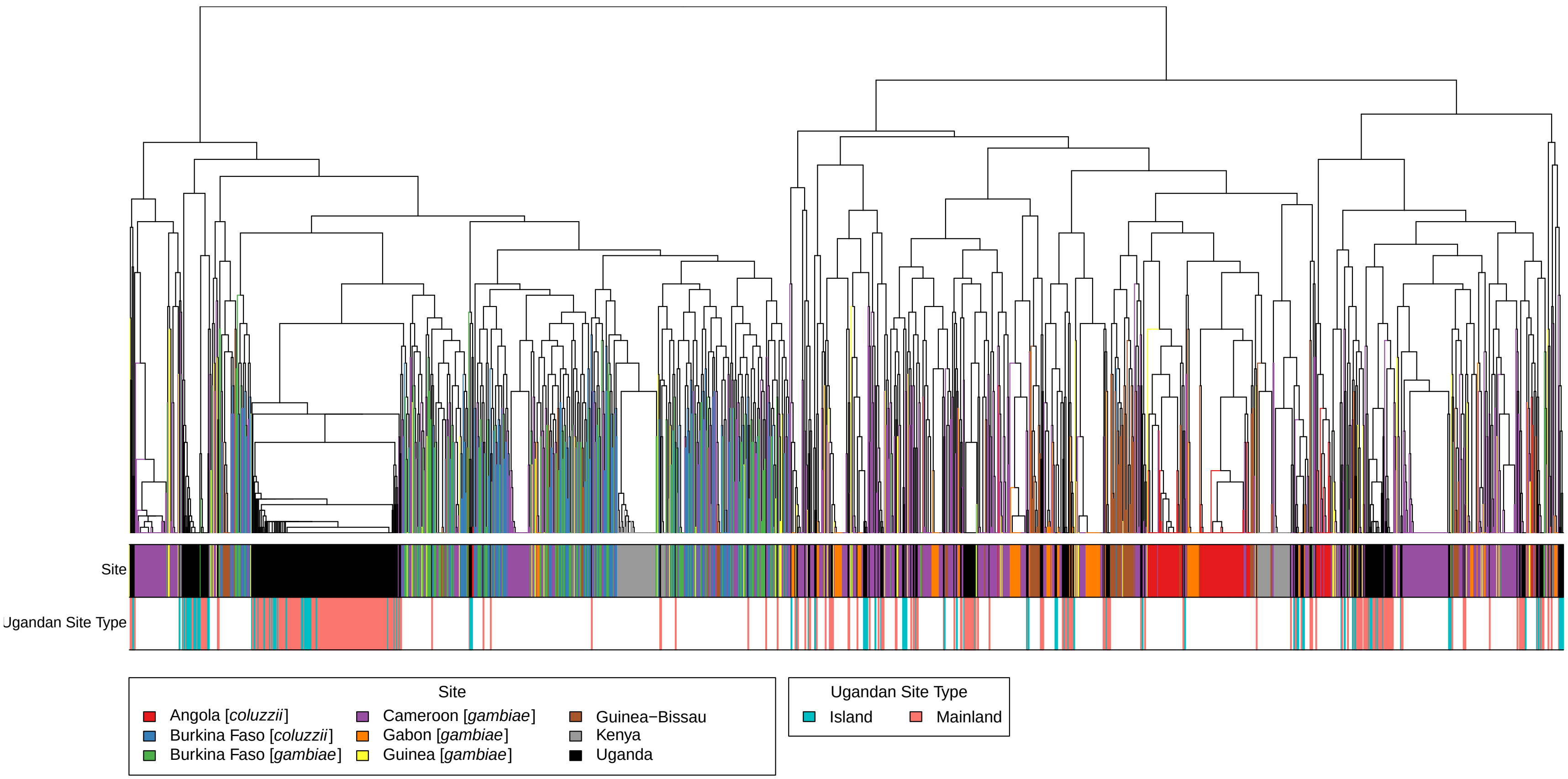

Figure S14: Tree for putative sweep on chromosome 2L.

Distance-based tree of haplotypes near putative sweep on chromosome 2L. Region shown is 10 kb up- and downstream of sweep target, centered at chr2L:34,044,820 (the approximate location of the peaks in pairwise statistics). Top color bar indicates locality, with all Ugandan individuals, from both the Ag1000G reference population and the LVB, in black. The bottom color bar differentiates the Ugandan individuals into mainland (red) and island (blue) individuals.

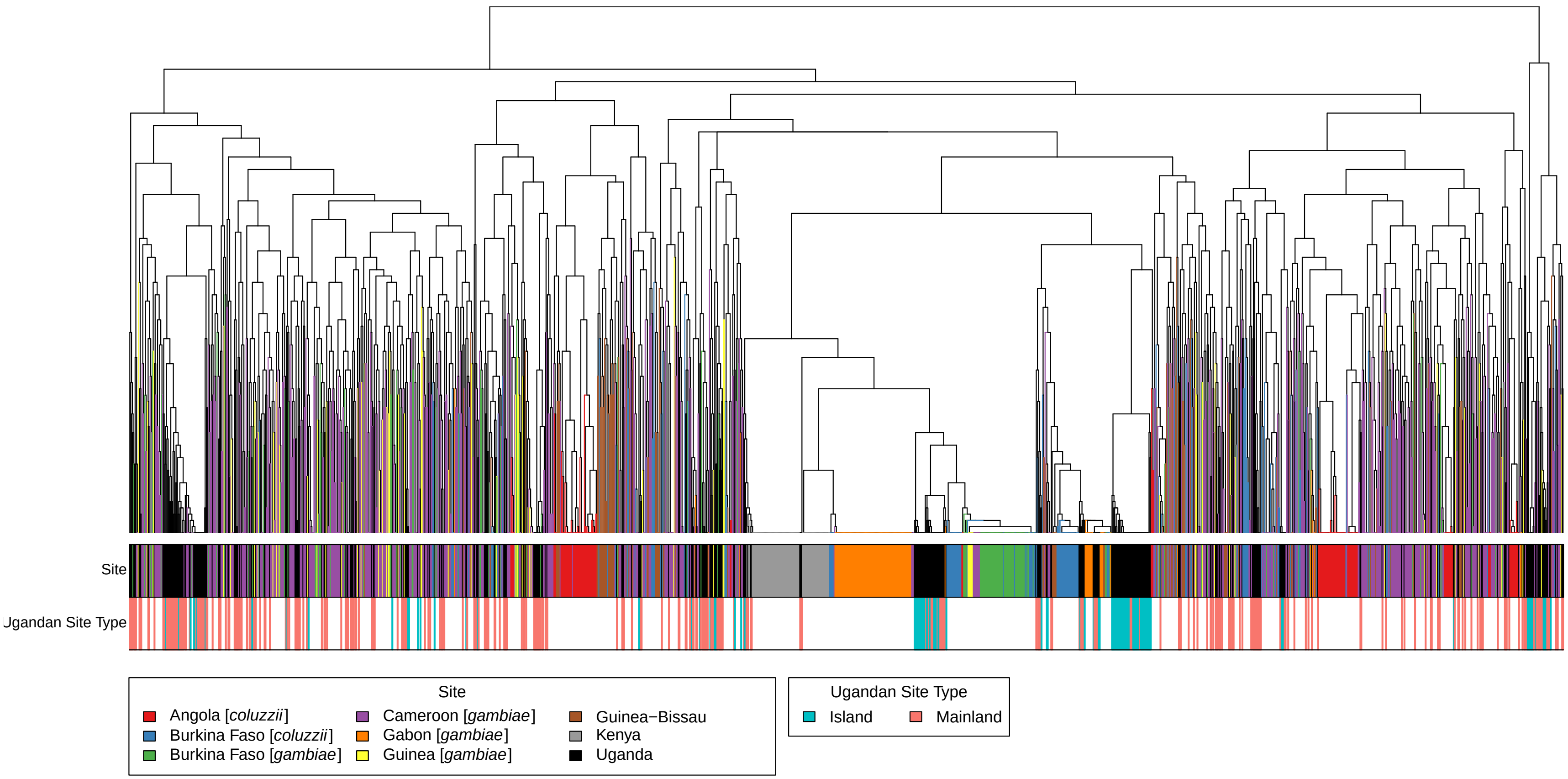

Figure S15: Tree for putative sweep on X-chromosome near *rdgA* ortholog.

Distance-based tree of haplotypes near putative sweep on X-chromosome. Region shown is 10 kb up- and downstream of sweep target, centered at chrX:9,238,942 (the approximate location of the peaks in pairwise statistics). Top color bar indicates locality, with all Ugandan individuals, from both the Ag1000G reference population and the LVB, in black. The bottom color bar differentiates the Ugandan individuals into mainland (red) and island (blue) individuals.

Literature Cited

Alexander, D., Novembre, J., & Lange, K. (2009). Fast model-based estimation of ancestry in unrelated individuals. *Genome Research*, *19*, 1655–1664. doi: 10.1101/gr.094052.109

Aronesty, E. (2011). *ea-utils: Command-line tools for processing biological sequencing data.*

Chang, C. C., Chow, C. C., Tellier, L. C., Vattikuti, S., Purcell, S. M., & Lee, J. J. (2015). Second-generation PLINK: rising to the challenge of larger and richer datasets. *GigaScience*, *4*(1), 7. doi: 10.1186/s13742-015-0047-8

Coffman, A. J., Hsieh, P. H., Gravel, S., & Gutenkunst, R. N. (2016). Computationally efficient composite likelihood statistics for demographic inference. *Molecular Biology and Evolution*, *33*(2), 591–593. doi: 10.1093/molbev/msv255

Danecek, P., Auton, A., Abecasis, G., Albers, C. A., Banks, E., DePristo, M. A., ... Durbin, R. (2011). The variant call format and VCFtools. *Bioinformatics*, *27*(15), 2156–8. doi: 10.1093/bioinformatics/btr330

Delaneau, O., Howie, B., Cox, A. J., Zagury, J. F., & Marchini, J. (2013). Haplotype estimation using sequencing reads. *American Journal of Human Genetics*, *93*(4), 687–696. doi: 10.1016/j.ajhg.2013.09.002

DePristo, M. A., Banks, E., Poplin, R., Garimella, K. V., Maguire, J. R., Hartl, C., ... Daly, M. J. (2011). A framework for variation discovery and genotyping using next-generation DNA sequencing data. *Nature Genetics*, *43*(5), 491–498. doi: 10.1038/ng.806

Galili, T. (2015). dendextend: an R package for visualizing, adjusting, and comparing trees of hierarchical clustering. *Bioinformatics*. doi: 10.1093/bioinformatics/btv428

Gautier, M., & Vitalis, R. (2012). rehh: An R package to detect footprints of selection in genome-wide SNP data from haplotype structure. *Bioinformatics*, *28*(8), 1176–1177.

Gutenkunst, R. N., Hernandez, R. D., Williamson, S. H., & Bustamante, C. D. (2009). Inferring the joint demographic history of multiple populations from multidimensional SNP frequency data. *PLoS Genetics*, *5*(10), e1000695. doi: 10.1371/journal.pgen.1000695

Jombart, T., & Ahmed, I. (2011). adegenet 1.3-1: new tools for the analysis of genome-wide SNP data. *Bioinformatics*. doi: 10.1093/bioinformatics/btr521

Kopelman, N. M., Mayzel, J., Jakobsson, M., Rosenberg, N. A., & Mayrose, I. (2015). CLUMPAK: a program for identifying clustering modes and packaging population structure inferences across K. *Molecular Ecology Resources*, *15*(5), 1179–1191. doi: 10.1016/j.coviro.2015.09.001.Human

Li, H. (2011). Tabix: Fast retrieval of sequence features from generic TAB-delimited files. *Bioinformatics*, *27*(5), 718–719. doi: 10.1093/bioinformatics/btq671

Li, H., & Durbin, R. (2009). Fast and accurate short read alignment with Burrows-Wheeler transform. *Bioinformatics*, *25*(14), 1754–1760. doi: 10.1093/bioinformatics/btp324

Li, H., Handsaker, B., Wysoker, A., Fennell, T., Ruan, J., Homer, N., ... Durbin, R. (2009). The Sequence Alignment/Map format and SAMtools. *Bioinformatics*, *25*(16), 2078–9. doi: 10.1093/bioinformatics/btp352

Liu, X., & Fu, Y.-X. (2015). Exploring population size changes using SNP frequency spectra. *Nature Genetics*, *47*(5), 555–559. doi: 10.1038/ng.3254

Miles, A., Harding, N. J., Bottà, G., Clarkson, C. S., Antão, T., Kozak, K., ... Kwiatkowski, D. P. (2017). Genetic diversity of the African malaria vector *Anopheles gambiae*. *Nature*, *552*, 96–100. doi: 10.1038/nature24995

Neuwirth, E. (2014). *RColorBrewer: ColorBrewer Palettes.*

Paradis, E., Claude, J., & Strimmer, K. (2004). APE: analyses of phylogenetics and evolution in R language. *Bioinformatics*, *20*, 289–290.

Patterson, N., Price, A. L., & Reich, D. (2006). Population structure and eigenanalysis. *PLoS Genetics*, *2*(12), 2074–2093. doi: 10.1371/journal.pgen.0020190

Price, A., Patterson, N. J., Plenge, R. M., Weinblatt, M. E., Shadick, N. A., & Reich, D. (2006). Principal components analysis corrects for stratification in genome-wide association studies. *Nature Genetics*, *38*(8), 904–9. doi: 10.1038/ng1847

Purcell, S., Neale, B., Todd-Brown, K., Thomas, L., Ferreira, M. A. R., Bender, D., ... Sham, P. C. (2007). PLINK: a tool set for whole-genome association and population-based linkage analyses. *American Journal of Human Genetics*, *81*(3), 559–75. doi: 10.1086/519795

Quinlan, A. R., & Hall, I. M. (2010). BEDTools: a flexible suite of utilities for comparing genomic features. *Bioinformatics*, *26*(6), 841–2. doi: 10.1093/bioinformatics/btq033

Sharakhova, M. V., Hammond, M. P., Lobo, N. F., Krzywinski, J., Unger, M. F., Hillenmeyer, M. E., ... Collins, F. H. (2007). Update of the *Anopheles gambiae* PEST genome assembly. *Genome Biology*, *8*(1), R5. doi: 10.1186/gb-2007-8-1-r5

Szpiech, Z. A., & Hernandez, R. D. (2014). selscan: an efficient multithreaded program to perform EHH-based scans for positive selection. *Molecular Biology and Evolution*, *31*(10), 2824–2827. doi: 10.1093/molbev/msu211

Tange, O. (2011). GNU Parallel - The Command-Line Power Tool. *;login: The USENIX Magazine*, *36*(1), 42–47. doi: http://dx.doi.org/10.5281/zenodo.16303
